# *Fgf10-*CRISPR mosaic mutants demonstrate the gene dose-related loss of the accessory lobe and decrease in the number of alveolar type 2 epithelial cells in mouse lung

**DOI:** 10.1371/journal.pone.0240333

**Published:** 2020-10-15

**Authors:** Munenori Habuta, Akihiro Yasue, Ken-ichi T. Suzuki, Hirofumi Fujita, Keita Sato, Hitomi Kono, Ayuko Takayama, Tetsuya Bando, Satoru Miyaishi, Seiichi Oyadomari, Eiji Tanaka, Hideyo Ohuchi

**Affiliations:** 1 Department of Cytology and Histology, Okayama University Graduate School of Medicine, Dentistry and Pharmaceutical Sciences, Okayama, Japan; 2 Department of Orthodontics and Dentofacial Orthopedics, Institute of Biomedical Sciences, Tokushima University Graduate School, Tokushima, Japan; 3 Department of Mathematical and Life Sciences, Hiroshima University, Hiroshima, Japan; 4 Center for the Development of New Model Organisms, National Institute for Basic Biology, Okazaki, Aichi, Japan; 5 Department of Legal Medicine, Okayama University Graduate School of Medicine, Dentistry and Pharmaceutical Sciences, Okayama, Japan; 6 Division of Molecular Biology, Institute of Advanced Medical Sciences, Tokushima University, Tokushima, Japan; Center of Pediatrics, GERMANY

## Abstract

CRISPR/Cas9-mediated gene editing often generates founder generation (F0) mice that exhibit somatic mosaicism in the targeted gene(s). It has been known that *Fibroblast growth factor 10* (*Fgf10*)-null mice exhibit limbless and lungless phenotypes, while intermediate limb phenotypes (variable defective limbs) are observed in the *Fgf10*-CRISPR F0 mice. However, how the lung phenotype in the *Fgf10-*mosaic mutants is related to the limb phenotype and genotype has not been investigated. In this study, we examined variable lung phenotypes in the *Fgf10*-targeted F0 mice to determine if the lung phenotype was correlated with percentage of functional *Fgf10* genotypes. Firstly, according to a previous report, *Fgf10*-CRISPR F0 embryos on embryonic day 16.5 (E16.5) were classified into three types: type I, no limb; type II, limb defect; and type III, normal limbs. Cartilage and bone staining showed that limb truncations were observed in the girdle, (type I), stylopodial, or zeugopodial region (type II). Deep sequencing of the *Fgf10*-mutant genomes revealed that the mean proportion of codons that encode putative functional FGF10 was 8.3 ± 6.2% in type I, 25.3 ± 2.7% in type II, and 54.3 ± 9.5% in type III (mean ± standard error of the mean) mutants at E16.5. Histological studies showed that almost all lung lobes were absent in type I embryos. The accessory lung lobe was often absent in type II embryos with other lobes dysplastic. All lung lobes formed in type III embryos. The number of terminal tubules was significantly lower in type I and II embryos, but unchanged in type III embryos. To identify alveolar type 2 epithelial (AECII) cells, known to be reduced in the *Fgf10*-heterozygous mutant, immunostaining using anti-surfactant protein C (SPC) antibody was performed: In the E18.5 lungs, the number of AECII was correlated to the percentage of functional *Fgf10* genotypes. These data suggest the *Fgf10* gene dose-related loss of the accessory lobe and decrease in the number of alveolar type 2 epithelial cells in mouse lung. Since dysfunction of AECII cells has been implicated in the pathogenesis of parenchymal lung diseases, the *Fgf10*-CRISPR F0 mouse would present an ideal experimental system to explore it.

## Introduction

The recently developed CRISPR/Cas9 system provides a highly efficient means for editing the genomes of model and non-model organisms. This powerful tool can help elucidate pathophysiological mechanisms underlying various genetic diseases (reviewed in [1, 2]). CRISPR/Cas9 promises the possibility of an ultimate cure for genetic diseases by enabling replacement of mutated genes with normal alleles (reviewed in [3]); however, genome-edited founder mice often exhibit somatic mosaicism in the targeted gene, meaning more than two mutated alleles for that gene are mixed in the same mouse [4–6]. Such mosaicism may be undesirable when it complicates phenotypic analysis [7]. However, mosaic mice may help the study of genes whose constitutive mutations are lethal, or the study of interactions between mutant and normal cells in the same individual [8]. Furthermore, recent DNA sequencing advances have enabled us to identify genetic mosaicism even in phenotypically normal individuals, the pathological significance of which is still unclear (reviewed in [9, 10]).

Previously, we reported that somatic mosaicism of a homeobox gene *Pax6* mutation causes variable eye phenotypes correlated with its gene dosage [11]. In this study, we focused on the variable phenotypes of genome-edited *Fgf10* founder generation (F0) mice. The *Fgf10* gene is required for limb and lung formation; *Fgf10* null mutants suffer embryonic lethality at birth due to lung agenesis [12, 13]. Recent studies have shown that a fraction of *Fgf10* genome-edited F0 mice exhibits typical limbless and lungless phenotypes, and that the severity of the limbless phenotype depends on the *Fgf10* mutation rate [7, 14, 15]. However, the lung phenotype has not been described. In this study, we sought to determine the relationship between lung and limb phenotypes, and correlation of these phenotypes to putative functional FGF10 dosage.

## Materials and methods

Reagents and equipment used in this study are listed in S1 Table.

### Mice and ethical statement

The animal experimental design was approved by the Committee of Animal Experiments of Tokushima University, Tokushima, Japan, and by the Animal Care and Use Committee, Okayama University, Okayama, Japan (Permit numbers: T27-16, T30-8, OKU-2017404, OKU2018605). All surgeries were performed under reversible anesthesia with medetomidine-midazolam-butorphanol and atipamezole, and all efforts were made to minimize suffering. *In vitro* fertilized (IVF) eggs were prepared from male and female BDF1 (C57BL/6 x DBA2 F1) mice as previously reported [14]. CRISPR/Cas9 genome editing was performed on one-cell stage zygotes by microinjection (analysis on E16.5 embryos) with *Cas9* mRNA and sgRNA targeted at exon 3 of the *Fgf10* gene according to Yasue et al. (2014) [14] (S1 Fig). After culturing genome-edited zygotes until the two-cell stage, embryos were transferred to the uterine tube of foster mice (Jcl: MCH (ICR) strain developed by CLEA Japan, Inc. (Tokyo, Japan); Jcl for Japan clea, MCH for Multi-Cross Hybrid, and ICR for Institute of Cancer Research) and allowed to develop until E16.5. To obtain E18.5 F0 embryos, *Cas9* mRNA (100 ng/μL) and synthesized guide RNA (50 ng/μL) were electroporated into one-cell stage IVF eggs and further manipulation of embryos was performed according to Hashimoto et al. (2016) [7]. CRISPR-control embryos were obtained by electroporation of medium only into IVF eggs before processing them for sequencing and histological examination. To know developmental variations, the weight of E18.5 embryos was measured with a standard electronic scale.

### Enzyme mismatch cleavage assay

Approximately 25 μg genomic DNA were extracted from neck skin tissue (epidermis and dermis) of collected embryos using a Qiagen DNeasy Blood & Tissue Kit. The genomic region encompassing the CRISPR/Cas9 target site was amplified by polymerase chain reaction (PCR) using a primer set specific for the *Fgf10* gene (S3A Fig, S1 Table) [14]. The amplicons were processed for a mismatch cleavage assay using the Guide-it Mutation Detection Kit and the products were analyzed by 2% agarose gel electrophoresis (S3B Fig).

### DNA sequencing

To verify the presence of the target site mutation, single bands of PCR amplicons of approximately 500 bp derived from the collected embryos at E16.5 (neck skin tissues) were subjected to Sanger sequencing. The resulting DNA sequence waves were dissociated using a web tool for TIDER data analysis available at https://tide.nki.nl [16].

Deep sequencing analysis using DNA extracted from neck skin, lung, and/or limb dermal tissues (Table 1 for E18.5; neck skin only for E16.5 embryos in Table 3; neck skin only for additional E18.5 embryos in S3 Table) for the *Fgf10* target site was performed according to the previous report on Illumina MiSeq system [17]. Briefly, the *Fgf10* on-target site from each individual embryo was amplified using custom barcode primers shown in S1 Table. Library construction and sequencing were performed at the National Institute for Basic Biology (NIBB, Japan) and Bioengineering Lab (Kanagawa, Japan). The pooled sequence data was demultiplexed into each sample and analyzed subsequently using a web-based tool, CLiCKAR [17].

**Table 1:** Summary of *Fgf10*-CRISPR F0 embryos at E18.5. These embryos were genotyped by deep sequencing (NGS).

Type	Embryo No.	Forelimb		Hindlimb		Weight (gram)	Analysis*	Tissues for NGS	Chi-square test of NGS results (*p-*value)	Functional *Fgf10* genotype (%) in neck DNA	Functional *Fgf10* genotype (mean±SEM %) in neck DNA	Functional *Fgf10* genotype (%) in lung DNA	Functional *Fgf10* genotype (%) in limb DNA	Genotype identity between different DNA	Figure
		Left	Right	Left	Right										
I	#18	ー	ー	ー	ー	0.6303	collected for c-b staining	Neck	N.A.	9.27	3.4 ± 2.9	N.D.	N.D.		
	#19	ー	ー	ー	ー	0.6899	collected for c-b stainig	Neck	N.A.	0.50		N.D.	N.D.		
	#26	ー	ー	ー	ー	0.4682	C-b staining	Neck	N.A.	0.45		N.D.	N.D.		
II	#14	ー	Humerus only	ー	Short femur	0.5784	C-b staining	Neck	N.A.	32.96	N.A.	N.D.	N.D.		Fig 1J–1O
III	#11	Normal	Normal	Normal	Normal	0.7799	Lung qPCR	Limb, lung, neck	3E-29	67.75	65.8 ± 9.0	61.19	64.23	Not identical	
	#12					0.9111	Lung qPCR	Limb, lung	0.7515	N.D.		65.98	66.45	Identical	Fig 1P–1R
	#13					0.9699	Lung collected for qPCR	Limb, lung	4E-05	N.D.		82.59	84.77	Not identical	
	#15					0.9572	Lung collected for qPCR	Limb, lung, neck	3E-11	59.78		56.84	56.22	Not identical	
	#16					0.9254	Lung collected for qPCR	Limb, lung	N.A.**	N.D.		100.00	100.00	Identical	
	#17					0.7524	Lung qPCR	Limb, lung	3E-50	N.D.		70.11	64.00	Not identical	
	#20					1.0555	IHC (19.9 ± 0.7)	Limb, neck	2E-09	100.00***		N.D.	100.00***	Not identical***	Fig 6J–6M
	#21					0.7742	IHC (16.9 ± 0.8)	Limb, neck	0.176	56.92		N.D.	57.52	Identical	Fig 6J–6M
	#22					0.8757	IHC (9.8 ± 0.5)	Limb, neck	0.2711	28.92		N.D.	29.94	Identical	Fig 6B, 6E, 6H;Fig 6J–6M
	#23					0.9232	IHC (24.2 ± 0.8)	Limb, neck	N.A.**	100.00		N.D.	97.20	Identical	Fig 6J–6M
	#24					0.9976	IHC (18.8 ± 0.7)	Limb, neck	1E-26	73.06		N.D.	82.26	Not identical	Fig 6J–6M
	#25					0.9832	IHC (15.2 ± 1.4)	Limb, neck	0.1395	40.75		N.D.	40.16	Identical	Fig 6J–6M
Wild type	#2	Normal	Normal	Normal	Normal	0.7472	Lung qPCR	Limb, lung, neck	N.A.**	99.63	N.A.	100.00	100.00	Identical	

*SPC (+) cells/total cells (mean ± SEM %) is shown in parenthesis of IHC for Fig 6J and 6K. -, no limb; c-b staining, cartilage and bone staining; N.A., not applicable; N.A.

**, not applicable due to low expected frequency (<5); N.D., not done; qPCR, quantitative PCR.

***contains wild type genotype and in-frame genotype retaining nucleotides for Lys196 and His201.

### Correction and calculation of putative somatic mutation rate in the case of large insertions

PCR amplicons for deep sequencing were analyzed using TAPEStation 4200 system. When larger DNA bands were detected above the main bands, their approximate molecular size was recorded. “Peak Molarity” value for each DNA band was documented and the percentage of large insertions was estimated by calculating the total value. The putative somatic mutation rate for large insertions was calculated proportionally. In embryos #3 (type II, E16.5), #23 (type III, E18.5), #11_2 (type II, E18.5), and #22_2 (type III, E18.5), large insertions were detected and corrected accordingly.

### Cartilage and bone staining

After Cesarean dissection, E18.5 embryos, removed their skin and internal organs, were fixed in 95% ethanol and processed for whole mount skeletal staining with Alcian blue 8GX and Alizarin red S according to standard procedures [18].

### Histology and analysis of terminal tubules

Hematoxylin-eosin (HE) staining was performed on mouse sections according to standard procedures. Briefly, sections of 5 μm-thickness were made of the pectoral region of paraffin-embedded embryos and mounted on microscope slides. After deparaffinization and rehydration, sections were stained with Mayer’s hematoxylin solution and 0.125% eosin. Following staining, sections were washed, dehydrated, and mounted with DPX. After HE staining, photos were taken with a 10x objective lens for each embryonic lung (n = 3 for each type, at E16.5) (Fig 5E–5H) and tiled using ImageJ (https://imagej.nih.gov/ij/index.html)) (RRID:SCR_003070). Terminal tubules were scored in the tiled full-field image (n = 1 for each embryo). The area for the full-field was calculated by ImageJ and the number of terminal tubules per mm^2^ were obtained and further analyzed statistically.

### Immunohistochemistry and analysis of SPC-positive cells

Immunohistochemistry was performed on the 5 μm-thick deparaffinized sections using ImmPRESS Polymer Detection Kit for IHC. A rabbit polyclonal antibody against a synthetic peptide fragment of human prosurfactant protein C (SPC) (within residues 1–100) was used at 1:5,000 dilution. The specificity of the antibody for AECIIs in the mouse lung has been validated [19]. The public identifier from the Antibody Registry is RRID: AB_10674024. After immunohistochemistry, SPC-positive cells were scored in random portions of a section in eight photomicrographs using a 40x objective lens. The total number of 6627 cells on average was counted per sample. The results were presented as a ratio of the number of positive cells per total number of cells [20]. This method has a limitation, in which as *Fgf10* mutations have been known to affect epithelial tube formation and branching [21] and therefore the number of distal airway epithelial cells in total may be impacted, the accuracy of the data would be ensured by normalizing AECII cell number to total epithelial cell number.

### Microscopy and image processing

Images for sections were collected with a Nikon DS-Fi1 camera on a Leica DM5000B microscope. Embryos, skeletons, and intestines were observed and imaged using a Leica DFC310FX camera on a Leica M165FC stereomicroscope. Image manipulation such as levels and color balance adjustments, made to some images, and assembly of figures were performed with Adobe Photoshop CS6 Extended (RRID:SCR_014199).

### Reverse transcription quantitative polymerase chain reaction (RT-qPCR)

Dissected embryonic lungs (E18.5) were immersed in RNAlater at 4°C overnight and stored at -80°C until further use. Total RNA was extracted using NucleoZOL. Five micrograms of RNA were reverse-transcribed to cDNA with FastGene cDNA Synthesis 5x ReadyMix. RT-qPCR was performed in duplicate wells with BrightGreen 2X qPCR MasterMix-No Dye and LightCycler Nano System using primers as shown in S4 Table. The PCR conditions employed were according to the manufacturer’s protocol: 10 min at 95°C for enzyme activation, 40 cycles of 15 sec at 95°C (denaturation) and 60 sec at 60°C (annealing and extension). We performed no template negative control experiments, as well as melting curve analysis according to manufacturer’s instructions. Gene expression was normalized to a housekeeping gene, *Glyceraldehyde-3-phosophate dehydrogenase* (*Gapdh*). Relative mRNA levels were determined by the comparative Ct method (Winer et al. 1999). Error bars in S6 Fig show standard deviation and p values were calculated by Microsoft Excel (RRID:SCR_016137) and confirmed by WaveMetrics Igor Pro (RRID:SCR_000325).

### Morphometric analysis of the cecum

Wild type (n = 3), type I (n = 4), and type II (n = 2) dissected intestines containing the cecum, colons, and part of small intestines at E18.5 were photographed at the same magnification (S7 Fig). The length of each cecum was measured using ImageJ. Briefly, multi-points were set from the most proximal point abutting the small intestine and colon to the most distal cecum, approximately 16 to 18 points in the case of wild type cecum. The sum of distance between each point was regarded as the length of the cecum.

### Statistical analysis

Significance was determined by one-way analysis of variance (ANOVA) (Fig 4B), Dunnett’s test (Fig 5I), or two-tailed unpaired Student’s t-test (S5L and S7J Figs). Correlation analysis was done by calculating correlation coefficient (Fig 6J–6L, S5M Fig). Chi-square test was used for the comparison of deep sequencing data on DNA from two or three different tissues (Table 4). Data were presented as mean ± standard error of the mean (SEM). Values of p<0.05 were considered significant otherwise stated. Data analysis was essentially performed using Microsoft Excel except for Dunnett’s test, which was done using R (https://www.r-project.org) (RRID:SCR_001905).

## Results

### Generation of *Fgf10*-CRISPR founder embryos and their limb skeletal structures

**Fig 1:**
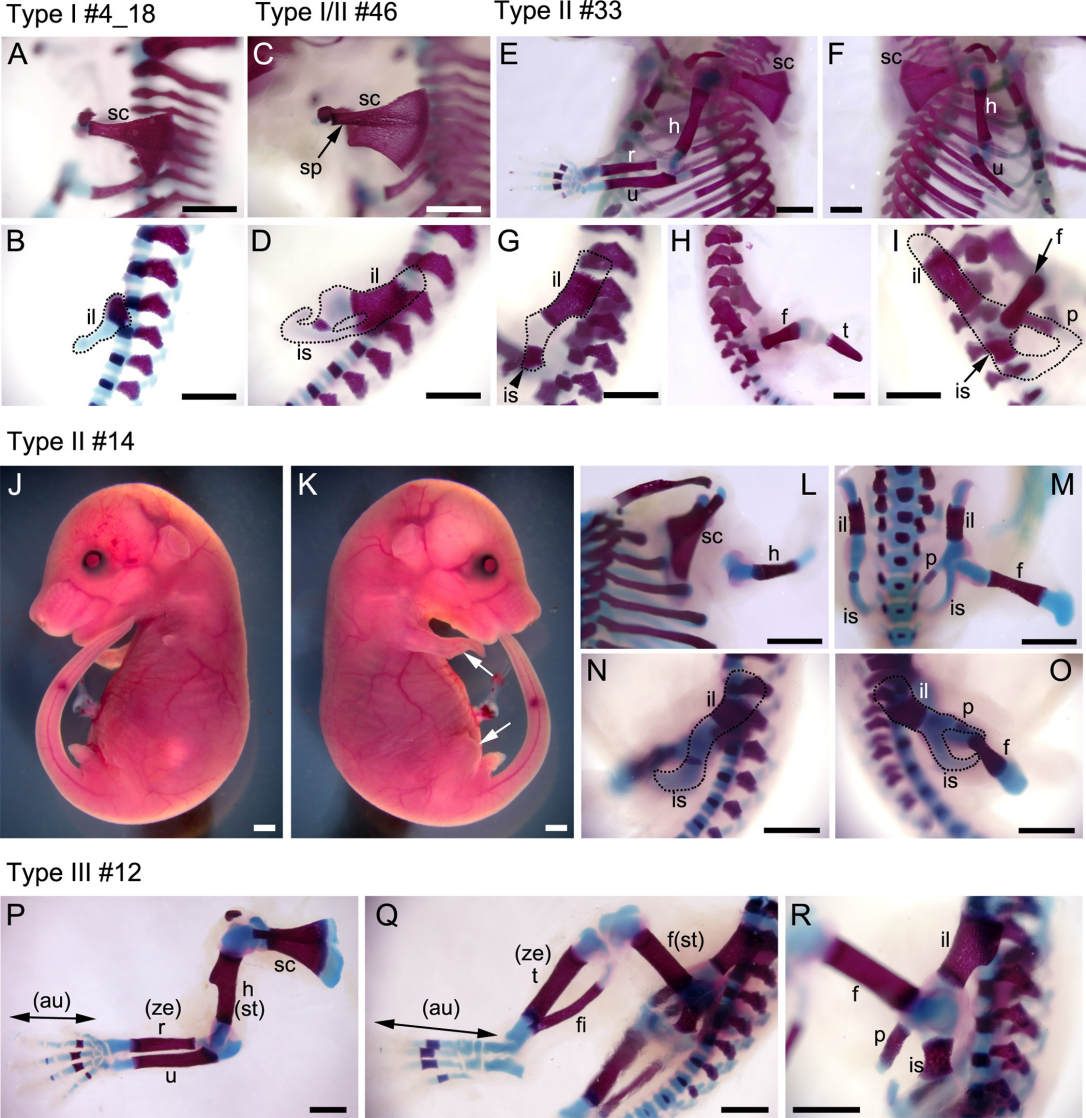
Cartilage and bone staining to reveal limb skeletal structures. **A-I, L-R**, left or right lateral views of limb and girdle regions (at E18.5) are shown. Cartilage is stained with Alcian blue and bone is stained with Alizarin red. **A, B**, type I #4_18 embryo. **C, D**, type I/II #46 embryo. **E-I**, type II #33 embryo. **L-O**, type II #14 embryo. **P-R**, type III #12 embryo. **J, K**, Lateral views of type II #14 embryo are shown. Arrows in (**K**) show truncated limbs. au, autopod; f, femur; fi, fibula; h, humerus; il, ilium; is, ischium; p, pubis; r, radius; sc, scapula; sp, spine; st, stylopod t, tibia; u, ulna; ze, zeugopod. Scale bars: 1 mm.

To generate *Fgf10*-CRISPR mice, we used a single guide RNA (sgRNA) to target exon 3, as previously described (S1 Fig) [14]. According to Hashimoto and Takemoto (2015) [15], the resultant *Fgf10*-CRISPR F0 embryos were classified by the limb phenotype into three types: type I, no limb; type II, limb defect; and type III, normal limbs. Since skeletal pattern of these three limb phenotypes has not been revealed, cartilage and bone staining was performed on E18.5 *Fgf10*-crispants (n = 4 for wild type; n = 11 for mutant embryos) (Table 2, Fig 1, S2 Fig). Normally, the appendicular skeleton consists of girdle (“pectoral” for forelimb and “pelvic” for hindlimb) and limb elements. The skeletons of the fore- and hindlimbs consist of stylopod for humerus/femur, zeugopod for the radius/ulna and tibia/fibula, and autopod for hand/foot (S2I–S2K Fig). In type I embryos (n = 6), all limb bones were lost and remaining girdle bones were affected to varying degrees (Fig 1A–1D; S2A–S2D Fig). The pectoral girdle consists of the scapula and clavicle, while the pelvic girdle consists of the ilium, pubis, and ischium (S2I–S2K Fig) (for review [22]). In four out of six type I embryos, the size of scapula was reduced and its spine was not formed, indicating that the posterior blade was lost (Fig 1A). As for the pelvic girdle, the ischial and pubic bones were lost, while iliac bones reduced bilaterally (Table 2 and Fig 1B). In the remaining two embryos, the scapula was intact with a spine, while the pubis and ischium were lost or reduced with the ilium intact bilaterally (Table 2 and Fig 1C and 1D; S2C and S2D Fig). Thus, we regarded the latter phenotype as a type between I and II (type I/II). In type II embryos (n = 2), all limb elements were lost in one or two appendages and in the remaining appendages, limb truncations were observed at the stylopod or zeugopod level (Table 2 and Fig 1E–1O; S2E and S2F Fig). In type III embryos examined (n = 3), all limb and girdle bone structures were normal (Fig 1P–1R). These limb skeletal patterns indicated that limb truncations were observed in the stylopodial, zeugopodial (type II), or pelvic region (type I). Formation of the clavicle, and the superior part of the scapula and pelvic girdle were not affected by lack of *Fgf10* gene products.

**Table 2:** Summary of *Fgf10*-CRISPR F0 embryos (E18.5) processed for cartilage and bone staining.

Type	Subtype	Embryo No.	Forelimb		Pectoral girdle				Hindlimb		Pelvic girdle						Functional *Fgf10* genotypes (%) in neck DNA	Functional *Fgf10* genotypes (%) in lung DNA	Functional *Fgf10* genotypes (%) in limb DNA	Figure
			Left	Right	Left		Right		Left	Right	Left			Right						
					Scaplua	Clavicle	Scaplua	Clavicle			Ilium	Ischium	Pubis	Ilium	Ischium	Pubis				
I	I	#4_18	-	-	±	+	±	+	-	-	±	-	-	±	-	-	N.A.	N.A.	N.A.	Fig 1,S2 Fig
		#26	-	-	±	+	±	+	-	-	±	-	-	±	-	-	0.45	N.A.	N.A.	
		#34	-	-	±	+	±	+	-	-	±	-	-	±	-	-	N.A.	N.A.	N.A.	
		#35	-	-	±	+	±	+	-	-	±	-	-	±	-	-	N.A.	N.A.	N.A.	
	I/II	#40	-	-	+	+	+	+	-	-	+	±	-	+	±	-	N.A.	N.A.	N.A.	
		#46	-	-	+	+	+	+	-	-	+	±	-	+	-	-	N.A.	N.A.	N.A.	Fig 1,S2 Fig
II		#14	N.D.	Humerus only	N.D.	N.D.	+	+	-	Truncated at distal femur	+	±	-	+	-	±	32.96	N.A.	N.A.	Fig 1
		#33	+	Humerus, radius only,no autopod	+	+	+	+	-	Truncated at distal tibia, no fibula	+	+	-	+	+	+	N.A.	N.A.	N.A.	Fig 1,S2 Fig
III		#11	+	+	+	N.D.	+	N.D.	+	+	+	+	+	+	+	+	67.75	61.19	64.23	
		#12	+	+	+	N.D.	+	N.D.	+	+	+	+	+	+	+	+	N.A.	65.98	66.45	Fig 1
		#17	+	+	+	N.D.	+	N.D.	+	+	+	+	+	+	+	+	N.A.	70.11	64.00	
Wild type		#1	+	+	+	N.D.	+	N.D.	+	+	+	+	+	+	+	+	N.A.	N.A.	N.A.	
		#4	+	+	+	N.D.	+	N.D.	+	+	+	+	+	+	+	+	N.A.	N.A.	N.A.	
		#5	+	+	+	N.D.	+	N.D.	+	+	N.D.	N.D.	N.D.	+	+	+	N.A.	N.A.	N.A.	
		#7_18	+	+	+	+	+	+	+	+	+	+	+	+	+	+	N.A.	N.A.	N.A.	Fig 1,S2 Fig

-, no bone; ±, bone hypoplasia; +, normal bones; N.A., not applicable; N.D., not determined due to separation of proximal parts of the limb from the trunk for lung analysis.

### Genomic analysis of *Fgf10*-CRISPR founder embryos

To prescreen mutations at the *Fgf10* locus, we performed an enzyme mismatch cleavage assay (S3A and S3B Fig) and Sanger sequencing with the use of DNA from neck skin tissues of the E16.5 embryos. We estimated mutation frequencies based on band intensities [23], and found an embryo (type II, #4) with obvious limb defects but showed faint band intensities for mutated DNA after endonuclease treatment (S3B Fig). Sanger sequencing indicated a mixed genotype of wild type and a single nucleotide insertion in this embryo (S3C Fig).

**Fig 2:**
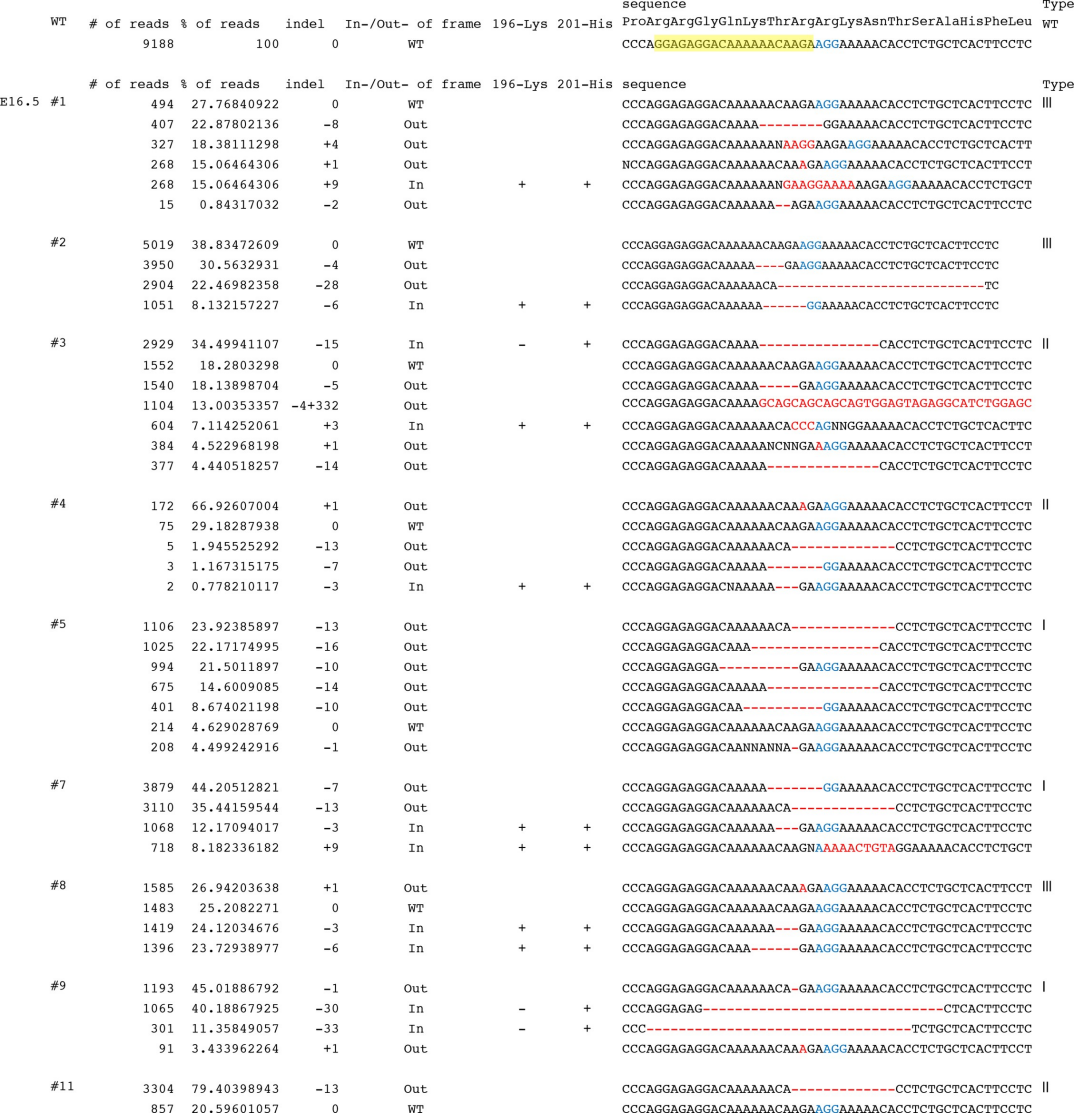
Genomic analysis of the *Fgf10*-CRISPR F0 embryos at E16.5 as revealed by deep sequencing. The target nucleotide sequence is highlighted in yellow. Proto-spacer adjacent motif sequence is shown in blue. Insertion and deletion sequences are shown in red.

**Fig 3:**
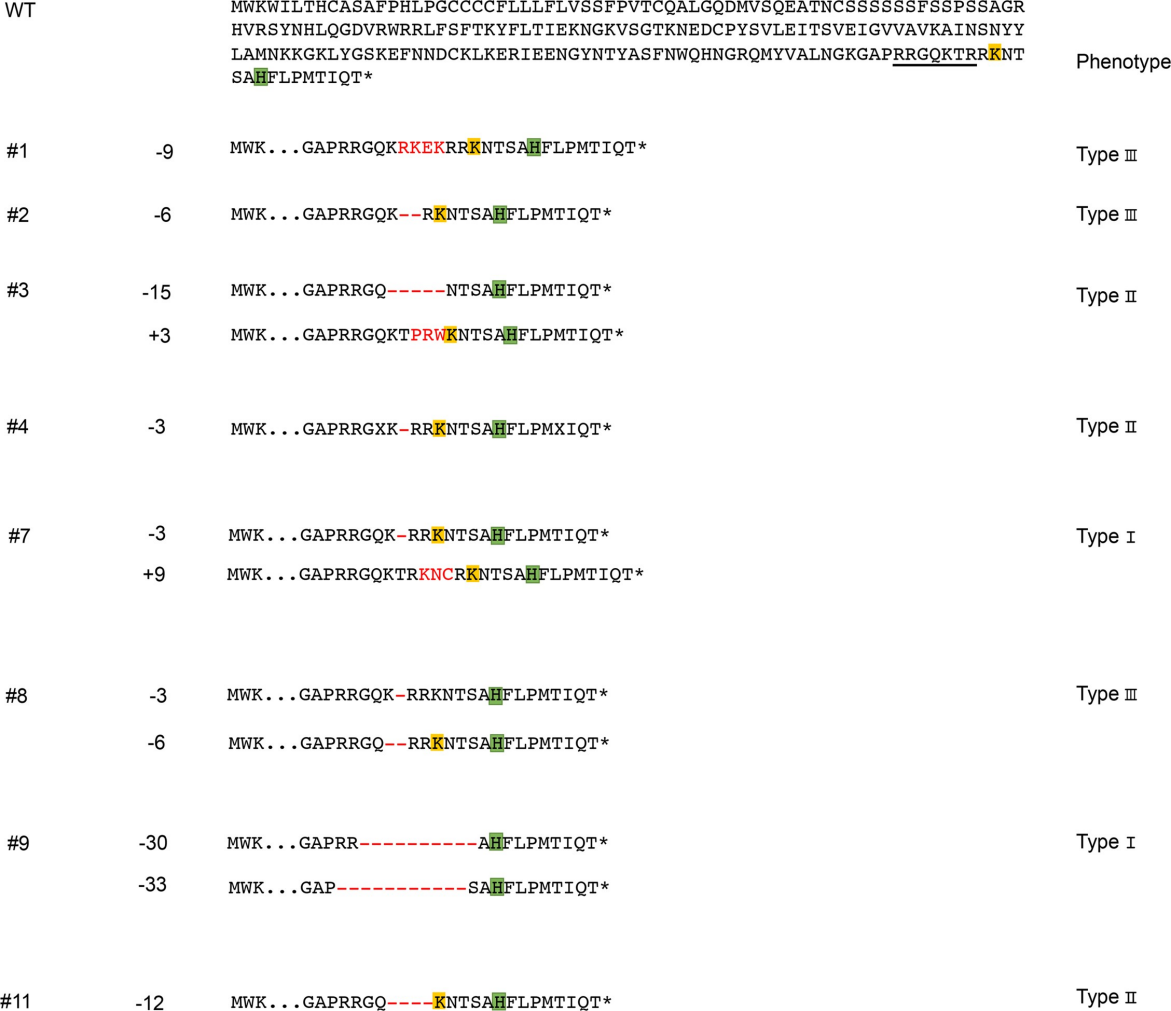
Deduced amino acids for in-frame mutations after deep sequencing. Wild type (WT) sequences are shown on the top. The embryo (E16.5) number (#), nucleotide number of small insertion (+) or deletion (-) (Indels) are shown on the left. Classified “types” are shown on the right. Amino acids corresponding to the guide RNA sequence are underlined. Deleted and altered amino acids are indicated in red.

**Fig 4:**
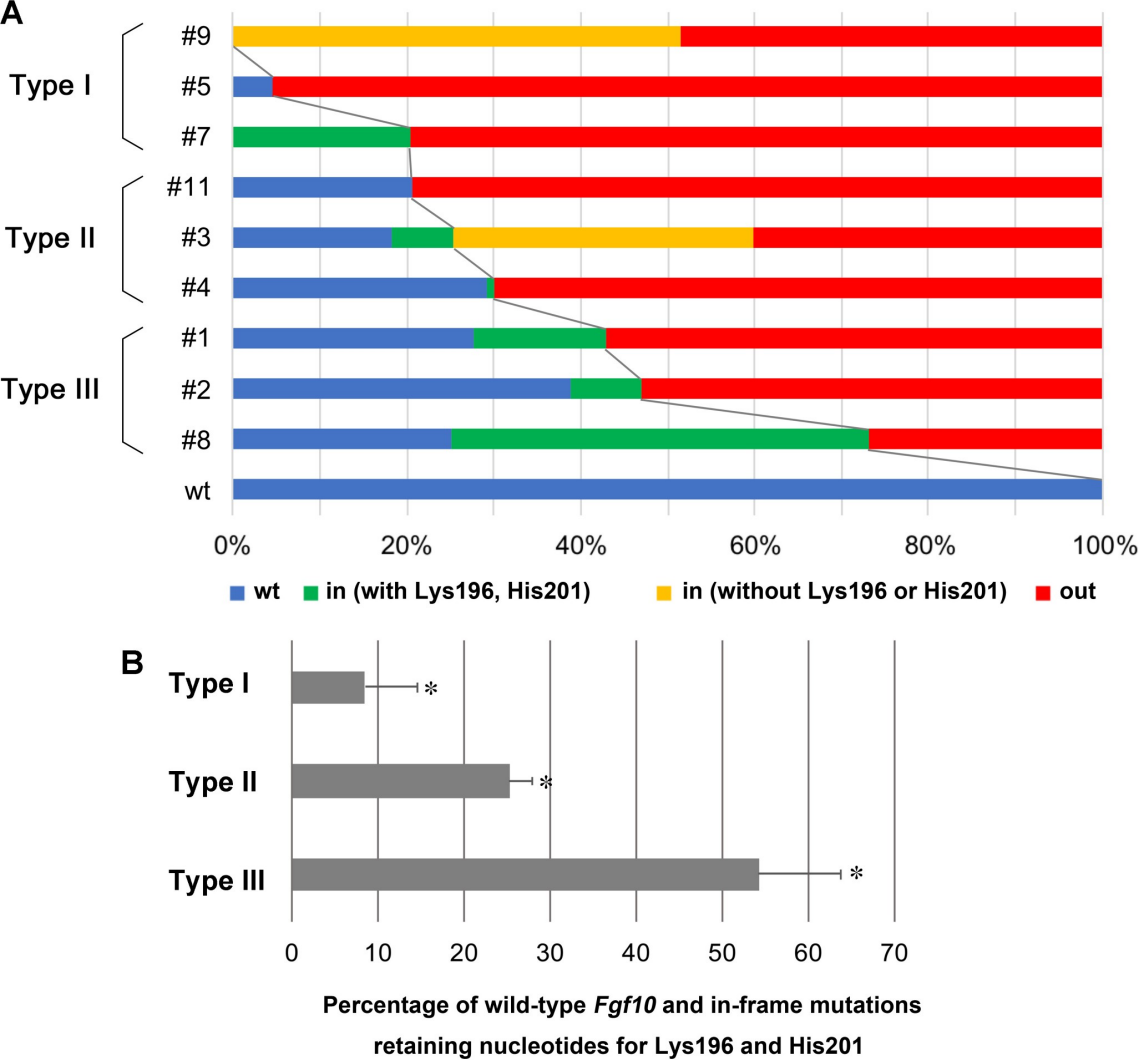
Schematic representation of genomic analysis of *Fgf10*-CRISPR F0 embryos at E16.5 by deep sequencing. **A**, percentage of total reads for the *Fgf10* crispants. wt, wild type *Fgf10* target nucleotide sequence; in, in-frame mutations by small insertion or deletion; out, frameshift mutations. Among in-frame mutations, the percentage of those that preserve the codons for Lys196 and His201 is shown in green, while the percentage that eliminate either of them or both is shown in yellow. **B**, percentage of wild type and in-frame mutations in which nucleotides for both Lys196 and His201 are retained: 8.3 ± 6.2% for type I, 25.3 ± 2.7% for type II, and 54.3 ± 9.5% for type III. Data are presented as means ± SEM. *p<0.01 (*p* = 0.008).

To get more accurate mutation frequencies, we performed deep sequencing of PCR amplicons containing the *Fgf10* target site by use of embryonic (E16.5) neck DNA. By multiple amplicon analysis, we could distinguish between wild type, in-frame and frameshift mutations (Fig 2). We also deduced amino acid sequences encoded by the targeted *Fgf10* locus (Fig 3). Among in-frame mutations, we found that codons that encode Lys196 and His201 were preserved or those that encode His201 alone were preserved as in #3, #5, and #9 embryos; both residues are known to be indispensable for FGF receptor dimerization [24]. We calculated the percentage of the gene sequence that still encodes putative functional FGF10 proteins with both residues in each type of mutant at E16.5, finding 8.3 ± 6.2% for type I, 25.3 ± 2.7% for type II, and 54.3 ± 9.5% for type III, n = 3 for each type, mean ± SEM) (Table 3) (Fig 4A and 4B). In the case of E18.5 neck DNA, it was 3.4 ± 2.9% for type I (n = 3), 33% for type II (n = 1), and 66.5 ± 8.8% for type III, respectively (n = 8) (Table 1).

**Table 3:** Summary of limb and lung phenotypes of *Fgf10-*CRISPR F0 embryos at E16.5.

Type	Embryo No.	Forelimb		Hindlimb		Lung					# of terminal tubules /mm2	# of terminal tubules/mm2 (mean ± SEM)	Functional Fgf10 genotype (%) (neck DNA)	Functional Fgf10 genotype (mean ± SEM %)	Figure
		Left	Right	Left	Right	Cranial lobe	Middle lobe	Caudal lobe	Accessory lobe	Left lobe					
#9	-	-	-	-	-	-	-	-	-	0	6.2 ± 6.2	0.00	8.3 ± 6.2	S4 Fig	
#5	-	-	-	-	-	-	-	-	-	0		4.63		S4 Fig	
#7	-	-	-	-	±*				-	18.7		20.35		Fig 5,S4 Fig	
II	#11	±	±	±	±	+	+	±	-	+	87.4	84.4 ± 13.9	20.60	25.3 ± 2.7	S4 Fig
	#3	±	±	±	±	+	-	+	-	+	59.0		25.39		Fig 5,S4 Fig
	#4	±	+	+	±	+	+	+	+	+	106.8		29.96		S4 Fig
III	#1	+	+	+	+	+	+	+	+	+	113.4	120.9 ± 4.0	42.83	54.3 ± 9.5	Fig 5,S4 Fig
	#2	+	+	+	+	+	+	+	+	+	127.1		46.97		S4 Fig
	#8	+	+	+	+	+	+	+	+	+	122.2		73.06		S4 Fig
WT	#1	+	+	+	+	+	+	+	+	+	131.2	135.6 ± 11.6	Not determined		Fig 5,S4 Fig
	#2	+	+	+	+	+	+	+	+	+	117.9				Not shown
	#3	+	+	+	+	+	+	+	+	+	157.6				Not shown

-, no limb or no lung lobe; ±, limb defect or small lung lobes; +, normal limbs or normal appearance of lung lobes.

*a residual lung lobe. Genotypes of crispants (types I-III) were prescreened by an enzyme mismatch cleavage assay (S3B Fig) and determined by deep sequencing (NGS).

To know whether genotyping results using DNA from different tissues were similar, we performed deep sequencing on DNA extracted from lung and limb dermal tissues as well as neck skin tissues of the E18.5 embryos (Table 1; S2 Table). We evaluated deep sequencing results on type III embryos (n = 10) with Chi-square test (Table 4). We found that in 6 embryos there was a significant difference in the frequencies of the functional *Fgf10* genotypes from the different tissues and in 4 embryos there was no significant difference in the frequencies of the functional *Fgf10* genotypes from the different tissues (Table 4).

**Table 4:** Chi-square test for genotyping results of DNA derived from different tissues.

Embryo No.	Type	Observed frequencies					Expected frequencies (EF)					*p-*value
#2	Wild type		Neck	Lung	Limb	Total		Neck	Lung	Limb	Total	N.A.
		Wt	4877	8760	58889	72526	Wt	4893.8	8757.8	58874	72526	
		Out	18	0	0	18	Out	0.0675	2.1736	15	18	
		Total	4895	8760	58889	72544	Total	4895	8760	58889	72544	
								EF: less than 5				
#11	III		Neck	Lung	Limb	Total		Neck	Lung	Limb	Total	3E-29
		Wt	6686	12893	18549	38128	Wt	6289.8	13430	18408	38128	
		Out	3182	8178	10331	21691	Out	3578.2	7640.6	10472	21691	
		Total	9868	21071	28880	59819	Total	9868	21071	28880	59819	
#12	III		Lung	Limb	Total			Lung	Limb	Total		0.7515
		Wt	706	19627	20333		Wt	710.8	19622	20333		
		Out	364	9911	10275		Out	359.2	9915.8	10275		
		Total	1070	29538	30608		Total	1070	29538	30608		
#13	III		Lung	Limb	Total			Lung	Limb	Total		4E-05
		Wt	4203	45331	49534		Wt	4304.1	45230	49534		
		Out	886	8147	9033		Out	784.89	8248.1	9033		
		Total	5089	53478	58567		Total	5089	53478	58567		
#15	III		Neck	Lung	Limb	Total		Neck	Lung	Limb	Total	3E-11
		Wt	7295	20266	22243	49804	Wt	6952.3	20313	22539	49804	
		W/o & Out	4908	15388	17318	37614	Out	5250.7	15341	17022	37614	
		Total	12203	35654	39561	87418	Total	12203	35654	39561	87418	
#16	III		Lung	Limb	Total			Lung	Limb	Total		N.A.
		Wt	15698	38223	53921		Wt	15698	38223	53921		
		Out	0	0	0		Out	0	0	0		
		Total	15698	38223	53921		Total	15698	38223	53921		
								EF: less than 5				
#17	III		Lung	Limb	Total			Lung	Limb	Total		3E-50
		Wt	3441	21706	25147		Wt	3505.1	21642	25147		
		In (w)	2869	13857	16726		In (w)	2331.3	14395	16726		
		Out	2690	20007	22697		Out	3163.6	19533	22697		
		Total	9000	55570	64570		Total	9000	55570	64570		
#20	III		Neck	Limb	Total			Neck	Limb	Total		2E-10
		Wt	29282	5549	34831		Wt	29043	5787.9	34831		
		In (w)	11875	2653	14528		In (w)	12114	2414.1	14528		
		Total	41157	8202	49359		Total	41157	8202	49359		
#21	III		Neck	Limb	Total			Neck	Limb	Total		0.176
		Wt	23077	10726	33803		Wt	23153	10650	33803		
		Out	17464	7923	25387		Out	17388	7998.7	25387		
		Total	40541	18649	59190		Total	40541	18649	59190		
#22	III		Neck	Limb	Total			Neck	Limb	Total		0.2711
		Wt	2653	963	3616		Wt	2677.4	938.58	3616		
		Out	6521	2253	8774		Out	6496.6	2277.4	8774		
		Total	9174	3216	12390		Total	9174	3216	12390		
#23	III		Neck	Limb	Total			Neck	Limb	Total		N.A.
*including large deletion		Wt	82324	1146	83470		Wt	82291	1178.5	83470		
		Out*	0	33	33		Out*	32.534	0.4659	33		
		Total	82324	1179	83503		Total	82324	1179	83503		
									EF: less than 5			
#24	III		Neck	Limb	Total			Neck	Limb	Total		1E-26
		Wt	8862	2633	11495		Wt	9094.9	2400.1	11495		
		Out	3268	568	3836		Out	3035.1	800.93	3836		
		Total	12130	3201	15331		Total	12130	3201	15331		
#25	III		Neck	Limb	Total			Neck	Limb	Total		0.1551
**including large insertion		In(w)	12665	10417	23082		In(w)	12582	10500	23082		
		Out**	18415	15520	33935		Out**	18498	15437	33935		
		Total	31080	25937	57017		Total	31080	25937	57017		

The number of sequence reads in deep sequencing is shown in contingency tables for observed frequencies. Wt, wild type *Fgf10* genotype; Out, frameshift mutations in the *Fgf10* gene; W/o & Out, in-frame mutation without nucleotides encoding Lys196 and/or His201 plus frameshift mutations in the *Fgf10* gene; In (w), in-frame mutations with nucleotides encoding Lys196 and His201; In (w/o), in-frame mutations without nucleotides encoding Lys196 and/or His201. When *p*-values <0.05, there is statistically significant difference in the *Fgf10* genotypes of the different tissues.

### Analysis of lung phenotypes

**Fig 5:**
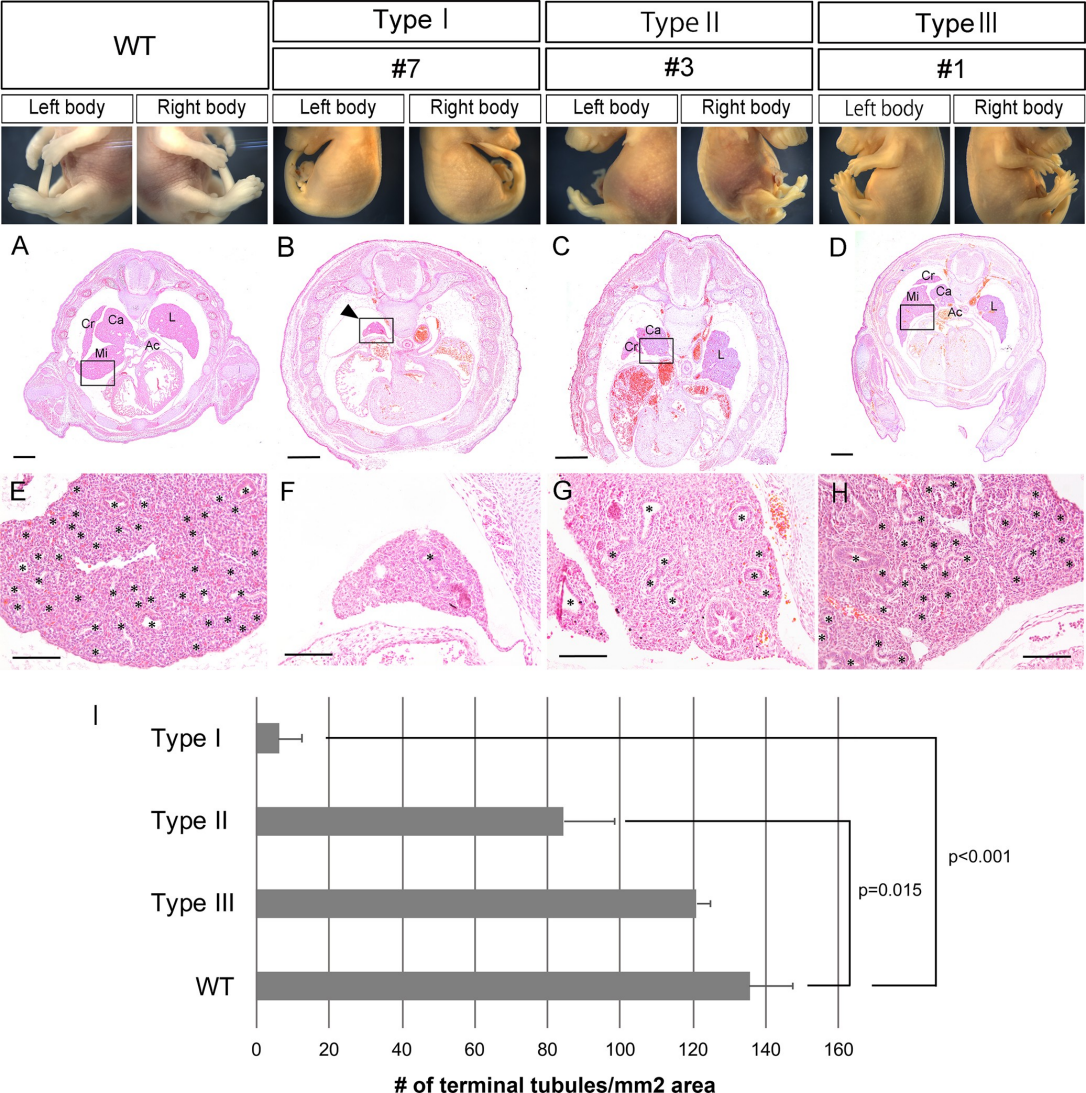
Limb phenotypes and lung histology of *Fgf10*-CRISPR F0 embryos at E16.5. Representative embryos are shown for wild type (WT), type I (embryo #7), type II (#3), and type III (#1). **A–D**, transverse section of the embryonic chest region. **E–H**, close-up of the embryonic lung (boxed area) shown in (**A–D**), respectively. Asterisks show putative terminal tubules in the lung. **I**, the number of lung terminal tubules per unit area. In type I and type II embryos, there is a significant decrease in the number compared with that of wild type. Data are presented as means ± SEM. Source data are available in Table 3. Ac, accessory lobe; Ca, caudal lobe; Cr, cranial lobe; L, left lobe; Mi, middle lobe. Scale bars: 500 μm in (**A–D**), and 100 μm in (**E–H**).

We next examined the lung histology of *Fgf10*-mosaic F0 embryos on E16.5 (Table 3, Fig 5, S4 Fig). In the normal mouse lung, there are five lung lobes, four on the right (cranial, middle, caudal, and accessory) and one on the left (Fig 5A). The lung histology of type I embryos demonstrated either the absence of all lobes (two of three embryos) or only the presence of a residual lobe (one of three) (Fig 5B). We found that the accessory lobe was absent in two out of three type II embryos, and that either middle or caudal lobe was additionally absent or hypoplastic (Fig 5C). In type III embryos, all five lung lobes were present (Fig 5D). In E18.5 type II embryos, the accessory lung lobe was absent from all three embryos examined (embryo #11_2, 24_2, 25_2; S5I–S5K Fig).

**Fig 6:**
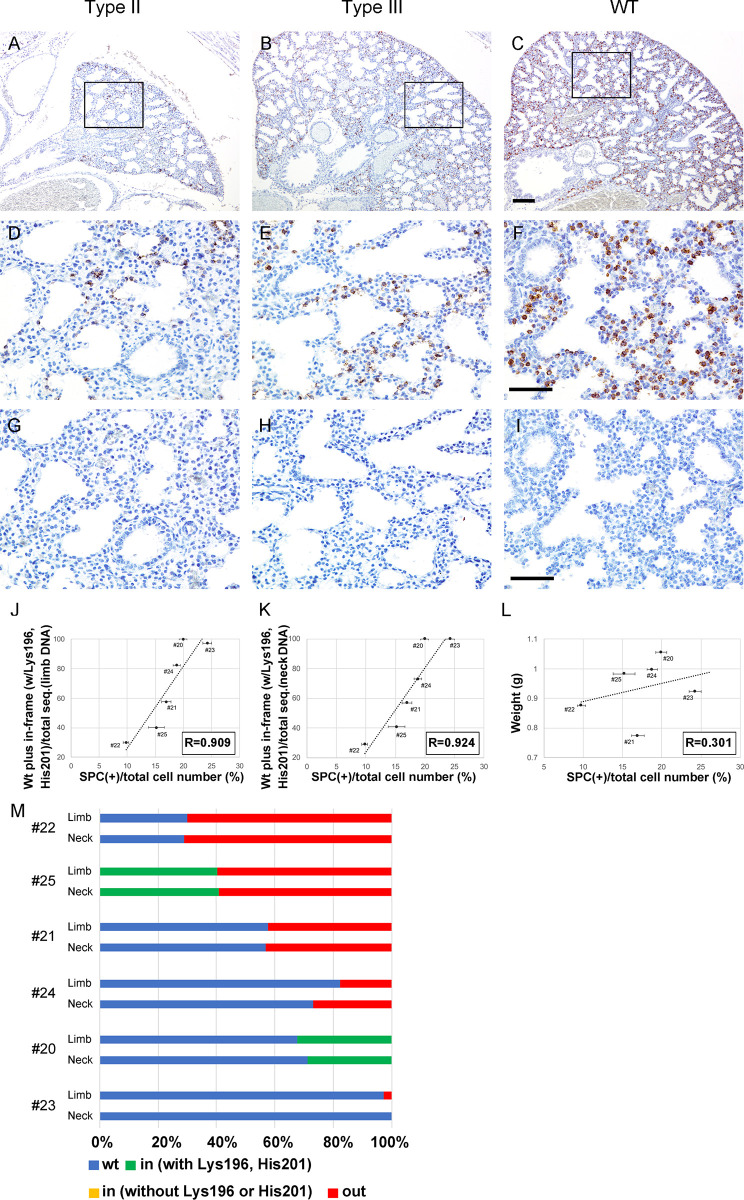
Immunohistochemistry of the lung in type II and type III *Fgf10*-crispants and wild type embryos at E18.5. Representative data are shown. Nuclei are stained with Hematoxylin. **A-C**, Localization of Surfactant protein C (SPC) (alveolar type 2 epithelial cells) is indicated by brown staining. **D-F**, close-up of the boxed area shown in (**A–C**), respectively. **G-I**, negative control, using normal rabbit IgG instead of anti-SPC antibody. Scale bars: 100 μm in (**A-C**); 50 μm in (**D-I**). **J, K**, the number of SPC-positive cells in type III embryos is correlated to the percentage of functional *Fgf10* genotypes in limb (**J**) or neck DNA (**K**). **L**, the number of SPC-positive cells in type III embryos is not to correlated to their weight (**K**). Source data for (**J-L**) are available in Table 1. **M**, Schematic drawings to show percentage of total reads for the type III *Fgf10* crispants. wt, wild type *Fgf10* target nucleotide sequence; in, in-frame mutations by small insertion or deletion; out, out-of-frame mutations. Among in-frame mutations, the percentage of those that preserve the codons for Lys196 and His201 is shown in green, while the percentage that eliminate either of them is shown in yellow.

Mouse lung development is divided into five stages, namely embryonic, pseudoglandular (E9.5 to 16.5), canalicular (E16.5 to 17.5), saccular (E17.5 to P5), and alveolar (P5 to P28) [25]. At E16.5, after branching morphogenesis the lung is in the transition of pseudoglandular to canalicular stage. We found that the number of terminal tubules was significantly decreased in type I and II residual lungs (Fig 5F, 5G and 5I), but not significantly altered in type III lungs (Fig 5H and 5I), compared with wild type (Fig 5E and 5I). By E18.5, the lung enters the saccular stage and cuboidal cells that produce surfactant protein C (SPC), i.e. alveolar type 2 epithelial (AECII) cells, differentiate on the wall of the developing airway space. Since AECII cell differentiation is perturbed in the *Fgf10-*heterozygous lung [26, 27], we performed immunohistochemistry on E18.5 lung sections to detect AECII. We counted the number of SPC-positive cells in lungs of type III embryos and examined whether it was correlated to the percentage of functional *Fgf10* genotype. Representative data of immunostaining for type III lungs are shown (Fig 6B and 6E) along with those for type II lungs (Fig 6A and 6D) and positive/negative controls (Fig 6C, 6F and 6G–6I). We found that the number of SPC-positive cells in lungs of type III embryos was correlated to the percentage of functional *Fgf10* genotype (Fig 6J, correlation coefficient is 0.909 for limb DNA; Fig 6K, 0.924 for neck DNA). Since the delay in development can be caused by variation between embryos even from a same litter due to crowding in the uterus, we also examined whether the number of SPC-positive cells was correlated to the weight of each embryo, as an index for developmental stage (Table 1). It seemed that there was no correlation between them (Fig 6L; correlation coefficient is 0.301).

On the other hand, since it was reported that the mesenchymal FGF10 is also important for proper mesenchymal lineage formation during lung development as revealed by *Fgf10*-hypomorphic lungs [20, 28], we examined expression of mesenchymal and its related marker genes [29] in type III lungs at E18.5 by quantitative PCR (Table 1, S4 Table). However, we could not detect a significant decrease in expression of these genes (S6 Fig), possibly because the percentage for functional *Fgf10* genotypes was too high to mimic a *Fgf10*-hypomorphic phenotype in lung mesenchymal lineage formation (61.19% in #11, 65.98% in #12, and 70.11% in #17 embryos). This expression analysis should be further clarified on earlier stage lungs, and in those which have more loss-of-function mutations in the *Fgf10* gene.

## Discussion

*Fgf10* genome edited F0 mice are classified into three limb phenotypes: no limb (type I), limb defect (type II), and normal limbs (type III). In this study, we first examined appendicular skeletal patterns of the *Fgf10* mosaic mutant mice by cartilage and bone staining. We found that varying degrees of limb and girdle bone truncations were observed in type I and type II embryos. We next estimated the rate of putative functional *Fgf10* genotypes in each mutant type and the mean percentage for type II embryos is 25.3 ± 2.7 by deep sequencing of neck DNA. Comparison of deep sequencing results on DNA from different tissues (neck, lung, and limb dermis) revealed that in half of the embryos the rate of putative functional *Fgf10* genotypes were significantly varied between the three DNA. Lung phenotypes were examined on two embryonic stages, E16.5 and E18.5. In type II embryos, the accessory lobe was lost and the number of terminal tubules was significantly reduced. In type II and “normal limb (type III)” embryos, the number of alveolar type 2 cells, immunostained by anti-surfactant C protein, was correlated with the rate of putative functional *Fgf10* genotypes.

### Truncation patterns of girdle and limb bones in *Fgf10*-mosaic mutants

Skeletal staining in this study revealed that in type II embryos, limb bone structures are truncated in more distal structures rather than forming a miniature of whole limb and girdle structures. Also, in the mutants, the zeugopodial element consists of one ulna- or tibia-like bone: This pattern is different from those after extirpation of apical ectodermal ridge in chicken limb buds, where both zeugopod bones are truncated [30]. Although the number of type II embryos examined in this study is only two and therefore further studies are needed, a reduced number of *Fgf10*-expressing cells in the mosaic mutants does not seem to know the morphological pattern to be formed. Looking the expression of zeugopod patterning genes could help to elucidate the underlying mechanisms.

Regarding girdle bones, it has been known that the posterior (inferior) half of the scapula is absent and most of the pelvic structures is lacking in *Fgf10*-null embryos [12, 13]. This study showed that in type I/II *Fgf10*-mosaic mutants the posterior scapula with the spine retained. In the mouse, while the pelvic girdle and most of the scapular blade derives solely from the lateral plate mesoderm (LPM), the medial edge of the blade arises from dermomyotome cells and the neural crest cells contribute to the scapular spine [31–33]. Thus, it is likely that a reduced number of *Fgf10*-expressing cells in the LPM and neural crest cells [34] contributed to the formation of the posterior (inferior) scapula in type I/II embryos. In the case of pelvic girdle, a reduced number of *Fgf10*-expressing cells in the LPM contributed to the formation of the inferior pelvic structures in type I/II embryos.

### *Fgf10* mutation rate may not be identical in different tissues

Here we showed that there is a correlation between limb phenotypes, lung phenotypes, and overall defective mutation rate in *Fgf10*-CRISPR knockout F0 mice. It has been shown that even if CRISPR-genome editing materials are introduced into fertilized eggs at the one-cell stage, mosaicism in the target genotype is caused under certain experimental conditions until the target sequences are mutated [6, 7]. During the early embryonic stage, mutated cells and non-mutated cells are intermingled and distributed to lung and limb primordia proportionally [35], which we believe contributes to the correlation between limb and lung phenotypes. However, statistical analysis on deep sequencing data of the genotypes shows in more than half cases there is a significant difference of the defective mutation rate in DNA from different tissues. In this study, we could not identify the reason why there were differences in the rate of the functional *Fgf10* genotypes. In some developmental settings, *Fgf10*-expressing normal cells might have a growth advantage than *Fgf10*-deficient cells, which should be further explored in the next studies [10].

### *Fgf10*-CRISPR F0 mice possess degrees of lung dysgenesis that correlate with putative functional FGF10 dosage

Accessory lobe formation was firstly impaired due to reduction in putative functional FGF10 dosage. Ramasamy et al. (2007) reported that *Fgf10*-hypomorphic mice, in which *Fgf10* expression is reduced by 27%, compared with *Fgf10*-heterozygous mutants, lack accessory lobe formation [20]. Since *Fgf10* expression is highest in the mesenchyme of the accessory lobe on E11.5 and persists as such until at least E18.5 [21, 29], high *Fgf10* dosage was believed to be required for accessory lobe formation. Our result shows that more than 25.3 ± 2.7% (from the analysis of E16.5 embryos) of functional *Fgf10* gene product is required for accessory lobe formation. Since reduction or loss of accessory lobe formation has been observed in loss-of-function mutants of *Gli3*, *Fog2*, and *Gata4* [36–38], FGF10 signaling may be related to the expression of these transcription factors during accessory lobe formation.

Lineage-tracing analysis of *Fgf10*-expressing cells during mouse lung development has revealed that there are two waves of *Fgf10* expression; the first begins from E11.5 and the second from E15.5 [29]. We think that a reduction in the latter cells would cause the decrease in the number of terminal tubules in type I and type II *Fgf10*-CRISPR F0 embryos. A recent study has shown that AECII as well as AECI independent progenitors are present at E13.5 as revealed by single cell RNA sequencing analysis [39]. By the saccular stage, the lung mesenchyme surrounding the epithelium becomes thinner and cuboidal AECII cells, characterized by the production of surfactant proteins, begin to differentiate (summarized in [40]). The number of AECII cells is found to be lower in the *Fgf10*-heterozygous lung [26, 27], which is similar to our result in type III *Fgf10*-mosaic mice. Taken together, *Fgf10* expression levels regulate the number of alveolar type 2 epithelial cells in mouse lung in somewhat dose-dependent manner.

Regarding FGF10 dosage, the *Fgf10*-hypomorphic mouse aforementioned [20, 28] has also clarified that higher FGF10 dosage is required for full development of colonic crypts [41]. Our preliminary observation (S7 Fig) showed that in type I embryos the length of the cecum was significantly shorter than the wild type and there was an atresia of the colon as reported in the *Fgf10*-null mutants [41, 42]. The length of the blind colon varied depending upon the embryos (S7D–S7G Fig), suggesting a correlation with their FGF10 dosage.

### *Fgf10*-CRISPR F0 mice can serve as a series of lung disease models

Volckaert et al. (2013) reported that lung branching morphogenesis does not require localized *Fgf10* expression in the distal mesenchyme, because ubiquitous *Fgf10* overexpression can induce lung formation in *Fgf10* knockout mice [43]. Furthermore, *Fgf10* expression after lung initiation is required for branching and proximal-distal differentiation by regulating *Sox2/9* expression in the epithelium [43]. Our result also supports this property of *Fgf10* as the number of terminal tubules decreased in the *Fgf10*-mosaic mutants where functional FGF10 dosage is reduced.

Recent papers have reported that mutations and single nucleotide polymorphisms (SNPs) in the *Fgf10* gene are correlated with human lung disease (reviewed in [44]). For example, the absence of the right medial-basal airway is associated with a type of chronic obstructive pulmonary diseases and two types of SNPs are found within the same intron of *Fgf10* in those cohorts [45]. Although some molecular and cellular differences have been identified between mouse and human lungs (reviewed in [46]), the F0 mice generated by CRISPR/Cas9-mediated *Fgf10* gene editing can become a model animal to study the pathophysiology of human pulmonary hypoplasia and related chronic lung diseases that may be rooted in the developmental stage as recently postulated [47]).

## Supporting information

S1 Table List of reagents and equipment used in this study. (DOCX)

S2 Table Summary of deep sequencing data on DNA from different tissues of E18.5 embryos. The percentage of sequence reads for each genotype category is shown in contingency tables. Wt, wild type *Fgf10* genotype; In, in-frame mutations in the *Fgf10* gene; Out, frameshift mutations in the *Fgf10* gene; N.D., not done; N.A., not applicable. *p*-values of Chi-square test (see Table 4) are shown for reference.
(DOCX)

S3 Table Summary of type II and type III embryos examined for E18.5 lungs by immunohistochemistry, shown in Fig 6 and S5 Fig. *Approximately 295- (#11_2) and 79- (#22_2) bp insertion were detected by microchip electrophoresis and the number of sequence reads in deep sequencing was corrected accordingly (see Materials and methods). N.D., not determined.
(DOCX)

S4 Table Primers used for quantitative PCR (qPCR) analysis. (DOCX)

S1 Fig Structure of the mouse *Fgf10* gene indicating the target sites for CRISPR/Cas9 system. (TIF)

S2 Fig Cartilage and bone staining to reveal skeletal structures. **A-H,** whole mount staining. Left (**A, C, E, G**) and right (**B, D, F, H**) lateral views are shown. Arrows show truncated limb and girdle bones. **I-K,** wild type skeletal structures, showing scapula and forelimb (**I**), hindlimb (**J**), and pelvic girdle (**K**). au, autopod; f, femur; fi, fibula; h, humerus; il, ilium; is, ischium; p, pubis; r, radius; sc, scapula; sp, spine; st, stylopod; t, tibia; u, ulna; ze, zeugopod. Scale bars: 1 mm.
(TIF)

S3 Fig Mismatch cleavage assay. **A**, PCR primers were designed in the upstream region of the exon 3 and in the exon 3, giving rise to a PCR amplicon size of 501 bp. DNA fragments of 309 bp and 192 bp are generated by the Resolvase when the *Fgf10* genome has been cleaved by Cas9 and non-homologous end joining has been achieved. **B**, Electrophoresis of the enzyme-treated mouse genomic DNA from the *Fgf10*-CRISPR F0 embryonic necks. The DNA ladder for DNA size reference and a result of DNA from a wild type (WT) mouse are shown on the left. Three DNA fragments of approximately 500 bp (▼), 300 bp (▽ in gray), and 200 bp (▽) are seen in all the lanes except for the wild type and #4 lanes. In embryo #3, an extra band for large insertion (328 base) is shown (see Fig 2). **C**, Genomic analysis of the #4 embryo as revealed by Sanger sequencing. Deduced amino acid sequences are also shown. Lys-196 and His-201 are highlighted in yellow and green, respectively. Altered amino acids are indicated in red. Asterisks indicate stop codons.
(TIF)

S4 Fig Limb phenotypes and lung histology of all embryos examined at E16.5 as summarized in Table 3. Scale bars: 500 μm.
(TIF)

S5 Fig Limb phenotypes, lung histology, and the number of SPC-positive cells per total cell number of type II and type III embryos at E18.5. Source data for (**L, M**) are available in S3 Table. Lateral views of type II (**A-F**), and type III (**G, H**) embryos at E18.5. Arrowheads show limb defects. (**I-K**) In all three type II embryos, the accessory lobe was lost. In embryo #24_2, (**J**), the middle lobe (Mi) was also undetectable. Data in (**L**) are presented as means ± SEM. (**M**) In these embryos, the number of SPC-positive cells was more correlated to the percentage of wild type *Fgf10* genotype (correlation coefficient [R] was 0.728 for neck DNA) than that including in-frame mutations with Lys196 and His201 retained (R = 0.334). Scale bar: 2 mm (**A-H**).
(TIF)

S6 Fig Quantitative PCR analysis for mRNA expression of mesenchymal and its related marker genes in wild type and type III lungs at E18.5. (TIF)

S7 Fig Cecum and colons of the wild type and *Fgf10*-CRISPR F0 (type I and type II) embryos at E18.5. **A-C**, wild type (Wt) cecum (c), colon, and small intestine (si) are shown from three embryos examined. Ileum and colon were cut at dissection. **D-G**, type I cecum is reduced compared with the wild type. Whether the cecum epithelium is absent or not cannot be identified from these photos. Type I embryos show an atresia of the colon, but the length varies depending upon the embryos. **H-I**, type II embryos examined (n = 2) do not exhibit a reduced cecum or an atresia of the colon. The colons presented here were cut as distally as possible. **J**, the approximate length of the cecum. The length of type I cecum is significantly decreased compared with the wild type. The length of two type II embryos examined is also shown for reference. Scale bar: 1 mm (in A for all to scale).
(TIF)

## References

[1] Doudna JA, Charpentier E. Genome editing. The new frontier of genome engineering with CRISPR-Cas9. Science. 2014;346(6213):1258096 10.1126/science.1258096 25430774

[2] Hsu PD, Lander ES, Zhang F. Development and applications of CRISPR-Cas9 for genome engineering. Cell. 2014;157(6):1262–78. 10.1016/j.cell.2014.05.010 24906146 PMC4343198

[3] Cornu TI, Mussolino C, Cathomen T. Refining strategies to translate genome editing to the clinic. Nat Med. 2017;23(4):415–23. 10.1038/nm.4313 28388605

[4] Sung YH, Kim JM, Kim HT, Lee J, Jeon J, Jin Y, et al Highly efficient gene knockout in mice and zebrafish with RNA-guided endonucleases. Genome Res. 2014;24(1):125–31. 10.1101/gr.163394.113 24253447 PMC3875853

[5] Yang H, Wang H, Shivalila CS, Cheng AW, Shi L, Jaenisch R. One-step generation of mice carrying reporter and conditional alleles by CRISPR/Cas-mediated genome engineering. Cell. 2013;154(6):1370–9. 10.1016/j.cell.2013.08.022 23992847 PMC3961003

[6] Yen ST, Zhang M, Deng JM, Usman SJ, Smith CN, Parker-Thornburg J, et al Somatic mosaicism and allele complexity induced by CRISPR/Cas9 RNA injections in mouse zygotes. Dev Biol. 2014;393(1):3–9. 10.1016/j.ydbio.2014.06.017 24984260 PMC4166609

[7] Hashimoto M, Yamashita Y, Takemoto T. Electroporation of Cas9 protein/sgRNA into early pronuclear zygotes generates non-mosaic mutants in the mouse. Dev Biol. 2016;418(1):1–9. 10.1016/j.ydbio.2016.07.017 27474397

[8] Zhong H, Chen Y, Li Y, Chen R, Mardon G. CRISPR-engineered mosaicism rapidly reveals that loss of Kcnj13 function in mice mimics human disease phenotypes. Sci Rep. 2015;5:8366 10.1038/srep08366 25666713 PMC4322368

[9] Biesecker LG, Spinner NB. A genomic view of mosaicism and human disease. Nat Rev Genet. 2013;14(5):307–20. 10.1038/nrg3424 23594909

[10] Gajecka M. Unrevealed mosaicism in the next-generation sequencing era. Mol Genet Genomics. 2016;291(2):513–30. 10.1007/s00438-015-1130-7 26481646 PMC4819561

[11] Yasue A, Kono H, Habuta M, Bando T, Sato K, Inoue J, et al Relationship between somatic mosaicism of Pax6 mutation and variable developmental eye abnormalities-an analysis of CRISPR genome-edited mouse embryos. Sci Rep. 2017;7(1):53 Epub 2017/03/02. 10.1038/s41598-017-00088-w 28246397 PMC5428340

[12] Min H, Danilenko DM, Scully SA, Bolon B, Ring BD, Tarpley JE, et al Fgf-10 is required for both limb and lung development and exhibits striking functional similarity to Drosophila branchless. Genes Dev. 1998;12(20):3156–61. 10.1101/gad.12.20.3156 9784490 PMC317210

[13] Sekine K, Ohuchi H, Fujiwara M, Yamasaki M, Yoshizawa T, Sato T, et al Fgf10 is essential for limb and lung formation. Nat Genet. 1999;21(1):138–41. 10.1038/5096 9916808

[14] Yasue A, Mitsui SN, Watanabe T, Sakuma T, Oyadomari S, Yamamoto T, et al Highly efficient targeted mutagenesis in one-cell mouse embryos mediated by the TALEN and CRISPR/Cas systems. Sci Rep. 2014;4:5705 10.1038/srep05705 25027812 PMC4099983

[15] Hashimoto M, Takemoto T. Electroporation enables the efficient mRNA delivery into the mouse zygotes and facilitates CRISPR/Cas9-based genome editing. Sci Rep. 2015;5:11315 10.1038/srep11315 26066060 PMC4463957

[16] Brinkman EK, Kousholt AN, Harmsen T, Leemans C, Chen T, Jonkers J, et al Easy quantification of template-directed CRISPR/Cas9 editing. Nucleic Acids Res. 2018;46(10):e58 10.1093/nar/gky164 29538768 PMC6007333

[17] Iida M, Suzuki M, Sakane Y, Nishide H, Uchiyama I, Yamamoto T, et al A simple and practical workflow for genotyping of CRISPR-Cas9-based knockout phenotypes using multiplexed amplicon sequencing. Genes Cells. 2020;00:1–12. 10.1111/gtc.12775 10.1111/gtc.12775 32323394

[18] Hogan B, Beddington R, Constantini F, Lacy E. Manipulating the mouse embryo: a laboratory manual. Cold Spring Harbor, NY: Cold Spring Harbor Laboratory Press; 1994.

[19] Stachtea XN, Tykesson E, van Kuppevelt TH, Feinstein R, Malmstrom A, Reijmers RM, et al Dermatan Sulfate-Free Mice Display Embryological Defects and Are Neonatal Lethal Despite Normal Lymphoid and Non-Lymphoid Organogenesis. PLoS One. 2015;10(10):e0140279 10.1371/journal.pone.0140279 26488883 PMC4619018

[20] Ramasamy SK, Mailleux AA, Gupte VV, Mata F, Sala FG, Veltmaat JM, et al Fgf10 dosage is critical for the amplification of epithelial cell progenitors and for the formation of multiple mesenchymal lineages during lung development. Dev Biol. 2007;307(2):237–47. 10.1016/j.ydbio.2007.04.033 17560563 PMC3714306

[21] Bellusci S, Grindley J, Emoto H, Itoh N, Hogan BL. Fibroblast growth factor 10 (FGF10) and branching morphogenesis in the embryonic mouse lung. Development. 1997;124(23):4867–78. 9428423 10.1242/dev.124.23.4867

[22] Sears KE, Capellini TD, Diogo R. On the serial homology of the pectoral and pelvic girdles of tetrapods. Evolution. 2015;69(10):2543–55. 10.1111/evo.12773 26374500

[23] Abu-Bonsrah KD, Zhang D, Newgreen DF. CRISPR/Cas9 Targets Chicken Embryonic Somatic Cells In Vitro and In Vivo and generates Phenotypic Abnormalities. Sci Rep. 2016;6:34524 10.1038/srep34524 27694906 PMC5046125

[24] Ibrahimi OA, Yeh BK, Eliseenkova AV, Zhang F, Olsen SK, Igarashi M, et al Analysis of mutations in fibroblast growth factor (FGF) and a pathogenic mutation in FGF receptor (FGFR) provides direct evidence for the symmetric two-end model for FGFR dimerization. Mol Cell Biol. 2005;25(2):671–84. 10.1128/MCB.25.2.671-684.2005 15632068 PMC543411

[25] Gao X, Hogan BL. Development of the Respiratory System Kaufman's Atlas of Mouse Development Supplement: With Coronal Sections: Academic Press; 2016.

[26] Chao CM, Yahya F, Moiseenko A, Tiozzo C, Shrestha A, Ahmadvand N, et al Fgf10 deficiency is causative for lethality in a mouse model of bronchopulmonary dysplasia. J Pathol. 2017;241(1):91–103. 10.1002/path.4834 27770432 PMC5164852

[27] Li J, Wang Z, Chu Q, Jiang K, Li J, Tang N. The Strength of Mechanical Forces Determines the Differentiation of Alveolar Epithelial Cells. Dev Cell. 2018;44(3):297–312 e5. 10.1016/j.devcel.2018.01.008 29408236

[28] Mailleux AA, Kelly R, Veltmaat JM, De Langhe SP, Zaffran S, Thiery JP, et al Fgf10 expression identifies parabronchial smooth muscle cell progenitors and is required for their entry into the smooth muscle cell lineage. Development. 2005; 132(9):2157–66. 10.1242/dev.01795 15800000

[29] El Agha E, Herold S, Al Alam D, Quantius J, MacKenzie B, Carraro G, et al Fgf10-positive cells represent a progenitor cell population during lung development and postnatally. Development. 2014;141(2):296–306. 10.1242/dev.099747 24353064 PMC3879811

[30] Summerbell D. A quantitative analysis of the effect of excision of the AER from the chick limb-bud. J Embryol Exp Morphol. 1974;32(3):651–60. 4463222

[31] Durland JL, Sferlazzo M, Logan M, Burke AC. Visualizing the lateral somitic frontier in the Prx1Cre transgenic mouse. J Anat. 2008;212(5):590–602. 10.1111/j.1469-7580.2008.00879.x 18430087 PMC2409079

[32] Valasek P, Theis S, Krejci E, Grim M, Maina F, Shwartz Y, et al Somitic origin of the medial border of the mammalian scapula and its homology to the avian scapula blade. J Anat. 2010;216(4):482–8. 10.1111/j.1469-7580.2009.01200.x 20136669 PMC2849525

[33] Matsuoka T, Ahlberg PE, Kessaris N, Iannarelli P, Dennehy U, Richardson WD, et al Neural crest origins of the neck and shoulder. Nature. 2005;436(7049):347–55. 10.1038/nature03837 16034409 PMC1352163

[34] Teshima TH, Lourenco SV, Tucker AS. Multiple Cranial Organ Defects after Conditionally Knocking Out Fgf10 in the Neural Crest. Front Physiol. 2016;7:488 10.3389/fphys.2016.00488 27826253 PMC5078472

[35] Soriano P, Jaenisch R. Retroviruses as probes for mammalian development: allocation of cells to the somatic and germ cell lineages. Cell. 1986;46(1):19–29. 10.1016/0092-8674(86)90856-1 3013418

[36] Grindley JC, Bellusci S, Perkins D, Hogan BL. Evidence for the involvement of the Gli gene family in embryonic mouse lung development. Dev Biol. 1997;188(2):337–48. 10.1006/dbio.1997.8644 9268579

[37] Ackerman KG, Herron BJ, Vargas SO, Huang H, Tevosian SG, Kochilas L, et al Fog2 is required for normal diaphragm and lung development in mice and humans. PLoS Genet. 2005;1(1):58–65. 10.1371/journal.pgen.0010010 16103912 PMC1183529

[38] Ackerman KG, Wang J, Luo L, Fujiwara Y, Orkin SH, Beier DR. Gata4 is necessary for normal pulmonary lobar development. Am J Respir Cell Mol Biol. 2007;36(4):391–7. 10.1165/rcmb.2006-0211RC 17142311 PMC1899327

[39] Frank DB, Penkala IJ, Zepp JA, Sivakumar A, Linares-Saldana R, Zacharias WJ, et al Early lineage specification defines alveolar epithelial ontogeny in the murine lung. Proc Natl Acad Sci U S A. 2019;116(10):4362–71. 10.1073/pnas.1813952116 30782824 PMC6410851

[40] Ornitz DM, Yin Y. Signaling networks regulating development of the lower respiratory tract. Cold Spring Harb Perspect Biol. 2012;4(5). 10.1101/cshperspect.a008318 PMC3331697 22550231

[41] Sala FG, Curtis JL, Veltmaat JM, Del Moral PM, Le LT, Fairbanks TJ, et al Fibroblast growth factor 10 is required for survival and proliferation but not differentiation of intestinal epithelial progenitor cells during murine colon development. Dev Biol. 2006;299(2):373–85. 10.1016/j.ydbio.2006.08.001 16956603

[42] Burns RC, Fairbanks TJ, Sala F, De Langhe S, Mailleux A, Thiery JP, et al Requirement for fibroblast growth factor 10 or fibroblast growth factor receptor 2-IIIb signaling for cecal development in mouse. Dev Biol. 2004;265(1):61–74. 10.1016/j.ydbio.2003.09.021 14697353

[43] Volckaert T, Campbell A, Dill E, Li C, Minoo P, De Langhe S. Localized Fgf10 expression is not required for lung branching morphogenesis but prevents differentiation of epithelial progenitors. Development. 2013;140(18):3731–42. 10.1242/dev.096560 23924632 PMC3754473

[44] Danopoulos S, Shiosaki J, Al Alam D. FGF Signaling in Lung Development and Disease: Human Versus Mouse. Front Genet. 2019;10:170 10.3389/fgene.2019.00170 30930931 PMC6423913

[45] Smith BM, Traboulsi H, Austin JHM, Manichaikul A, Hoffman EA, Bleecker ER, et al Human airway branch variation and chronic obstructive pulmonary disease. Proc Natl Acad Sci U S A. 2018;115(5):E974–E81. 10.1073/pnas.1715564115 29339516 PMC5798356

[46] Nikolic MZ, Sun D, Rawlins EL. Human lung development: recent progress and new challenges. Development. 2018;145(16). 10.1242/dev.163485 PMC6124546 30111617

[47] Hobbs BD, de Jong K, Lamontagne M, Bosse Y, Shrine N, Artigas MS, et al Genetic loci associated with chronic obstructive pulmonary disease overlap with loci for lung function and pulmonary fibrosis. Nat Genet. 2017;49(3):426–32. 10.1038/ng.3752 28166215 PMC5381275

