# Supplementary material for: Fgf10-CRISPR mosaic mutants demonstrate the gene dose-related loss of the accessory lobe and decrease in the number of alveolar type 2 epithelial cells in mouse lung
Source: PLoS One. 2020 Oct 15;15(10):e0240333. doi: 10.1371/journal.pone.0240333 (PMC7561199; doi:10.1371/journal.pone.0240333)
Supplement: S1 Table — (DOCX) [file pone.0240333.s001.docx]

**S1 Table. List of reagents and equipment used in this study.**
