# Supplementary material for: Fgf10-CRISPR mosaic mutants demonstrate the gene dose-related loss of the accessory lobe and decrease in the number of alveolar type 2 epithelial cells in mouse lung
Source: PLoS One. 2020 Oct 15;15(10):e0240333. doi: 10.1371/journal.pone.0240333 (PMC7561199; doi:10.1371/journal.pone.0240333)
Supplement: S2 Table — The percentage of sequence reads for each genotype category is shown in contingency tables. Wt, wild type Fgf10 genotype; In, in-frame mutations in the Fgf10 gene; Out, frameshift mutations in the Fgf10 gene; N.D., not done; N.A., not applicable. p-values of Chi-square test (see Table 4) are shown for reference. (DOCX) [file pone.0240333.s002.docx]

**S2 Table. Summary of deep sequencing data on DNA from different tissues of E18.5 embryos.**

**S2 Table (continued)**

The percentage of sequence reads for each genotype category is shown in contingency tables. Wt, wild type *Fgf10* genotype; In, in-frame mutations in the *Fgf10* gene; Out, frameshift mutations in the *Fgf10* gene; N.D., not done; N.A., not applicable. *p*-values of Chi-square test (see Table 4) are shown for reference.
