# Supplementary material for: Fgf10-CRISPR mosaic mutants demonstrate the gene dose-related loss of the accessory lobe and decrease in the number of alveolar type 2 epithelial cells in mouse lung
Source: PLoS One. 2020 Oct 15;15(10):e0240333. doi: 10.1371/journal.pone.0240333 (PMC7561199; doi:10.1371/journal.pone.0240333)
Supplement: S3 Table — *Approximately 295- (#11_2) and 79- (#22_2) bp insertion were detected by microchip electrophoresis and the number of sequence reads in deep sequencing was corrected accordingly (see Materials and methods). N.D., not determined. (DOCX) [file pone.0240333.s003.docx]

**S3 Table. Summary of type II and type III embryos examined for E18.5 lungs by immunohistochemistry, shown in Fig 6 and Fig S5.**

*Approximately 295- (#11_2) and 79- (#22_2) bp insertion were detected by microchip electrophoresis and the number of sequence reads in deep sequencing was corrected accordingly (see Materials and methods). N.D., not determined.
