# Supplementary figures and images for: Fgf10-CRISPR mosaic mutants demonstrate the gene dose-related loss of the accessory lobe and decrease in the number of alveolar type 2 epithelial cells in mouse lung
Source: PLoS One. 2020 Oct 15;15(10):e0240333. doi: 10.1371/journal.pone.0240333 (PMC7561199; doi:10.1371/journal.pone.0240333)

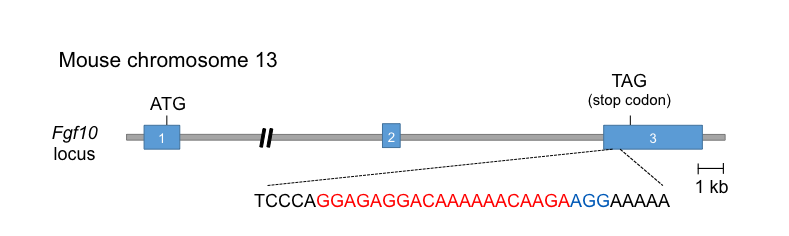

Supplement: S1 Fig — (TIF) [file pone.0240333.s005.tif]

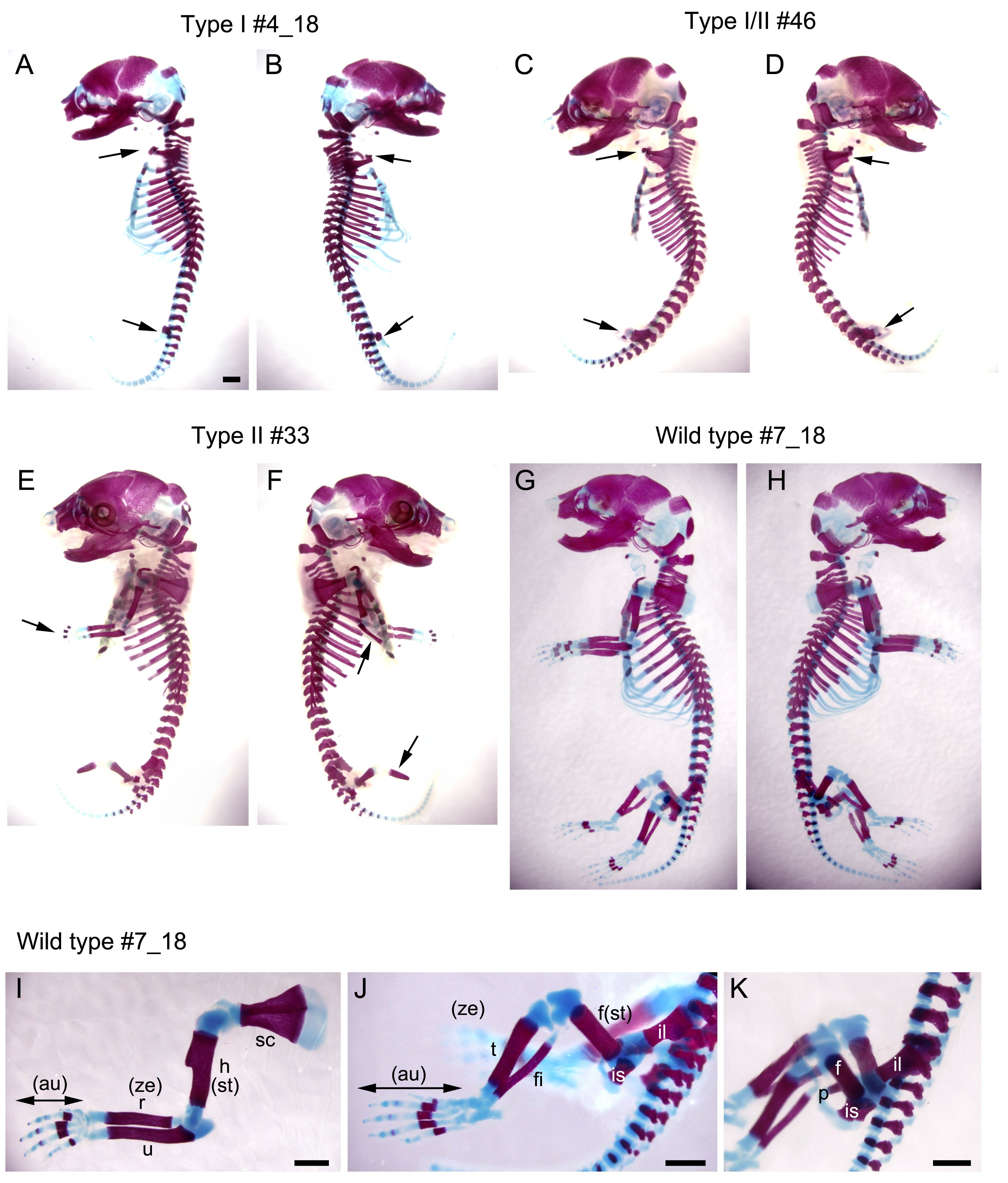

Supplement: S2 Fig — A-H, whole mount staining. Left (A, C, E, G) and right (B, D, F, H) lateral views are shown. Arrows show truncated limb and girdle bones. I-K, wild type skeletal structures, showing scapula and forelimb (I), hindlimb (J), and pelvic girdle (K). au, autopod; f, femur; fi, fibula; h, humerus; il, ilium; is, ischium; p, pubis; r, radius; sc, scapula; sp, spine; st, stylopod; t, tibia; u, ulna; ze, zeugopod. Scale bars: 1 mm. (TIF) [file pone.0240333.s006.tif]

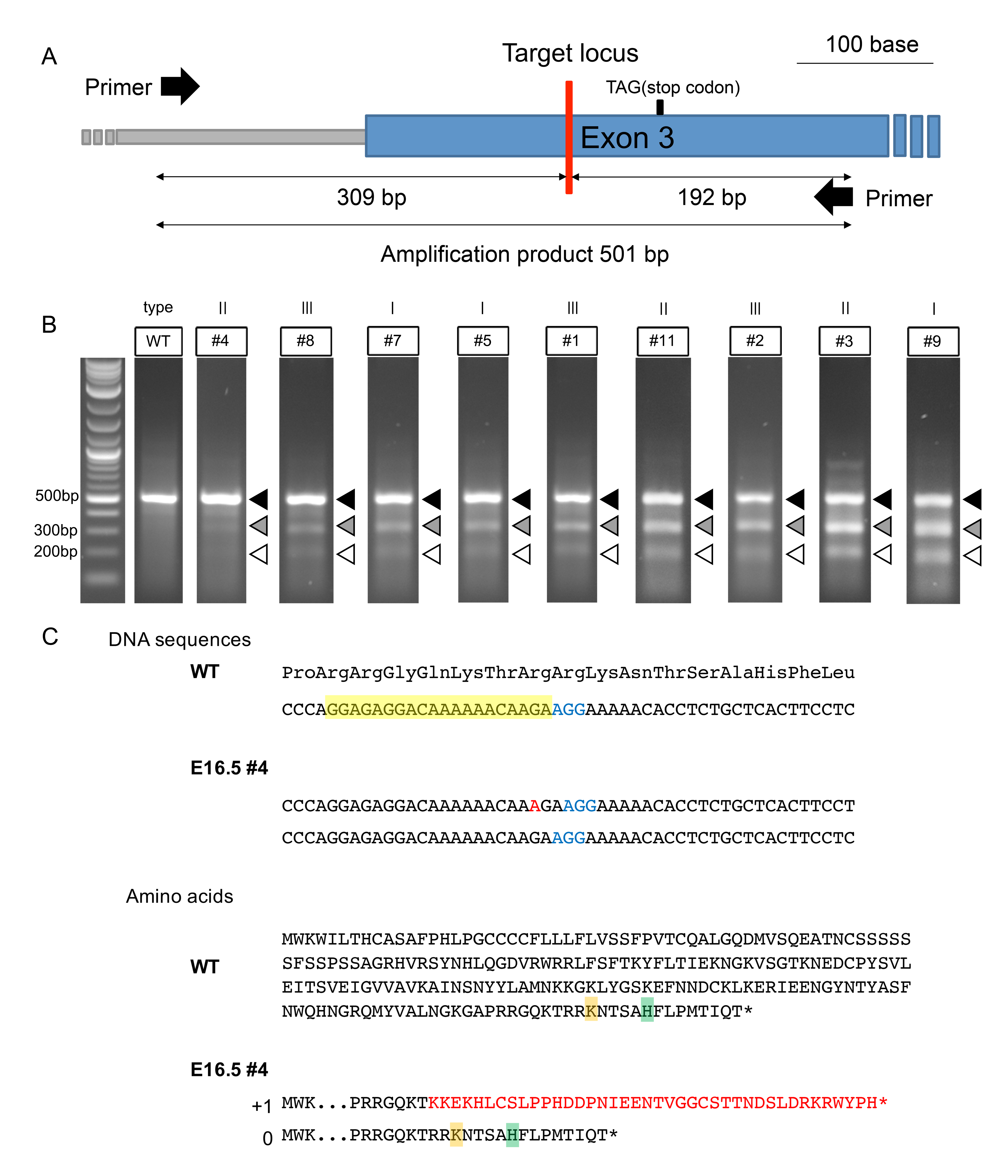

Supplement: S3 Fig — A, PCR primers were designed in the upstream region of the exon 3 and in the exon 3, giving rise to a PCR amplicon size of 501 bp. DNA fragments of 309 bp and 192 bp are generated by the Resolvase when the Fgf10 genome has been cleaved by Cas9 and non-homologous end joining has been achieved. B, Electrophoresis of the enzyme-treated mouse genomic DNA from the Fgf10-CRISPR F0 embryonic necks. The DNA ladder for DNA size reference and a result of DNA from a wild type (WT) mouse are shown on the left. Three DNA fragments of approximately 500 bp (▼), 300 bp (▽ in gray), and 200 bp (▽) are seen in all the lanes except for the wild type and #4 lanes. In embryo #3, an extra band for large insertion (328 base) is shown (see Fig 2). C, Genomic analysis of the #4 embryo as revealed by Sanger sequencing. Deduced amino acid sequences are also shown. Lys-196 and His-201 are highlighted in yellow and green, respectively. Altered amino acids are indicated in red. Asterisks indicate stop codons. (TIF) [file pone.0240333.s007.tif]

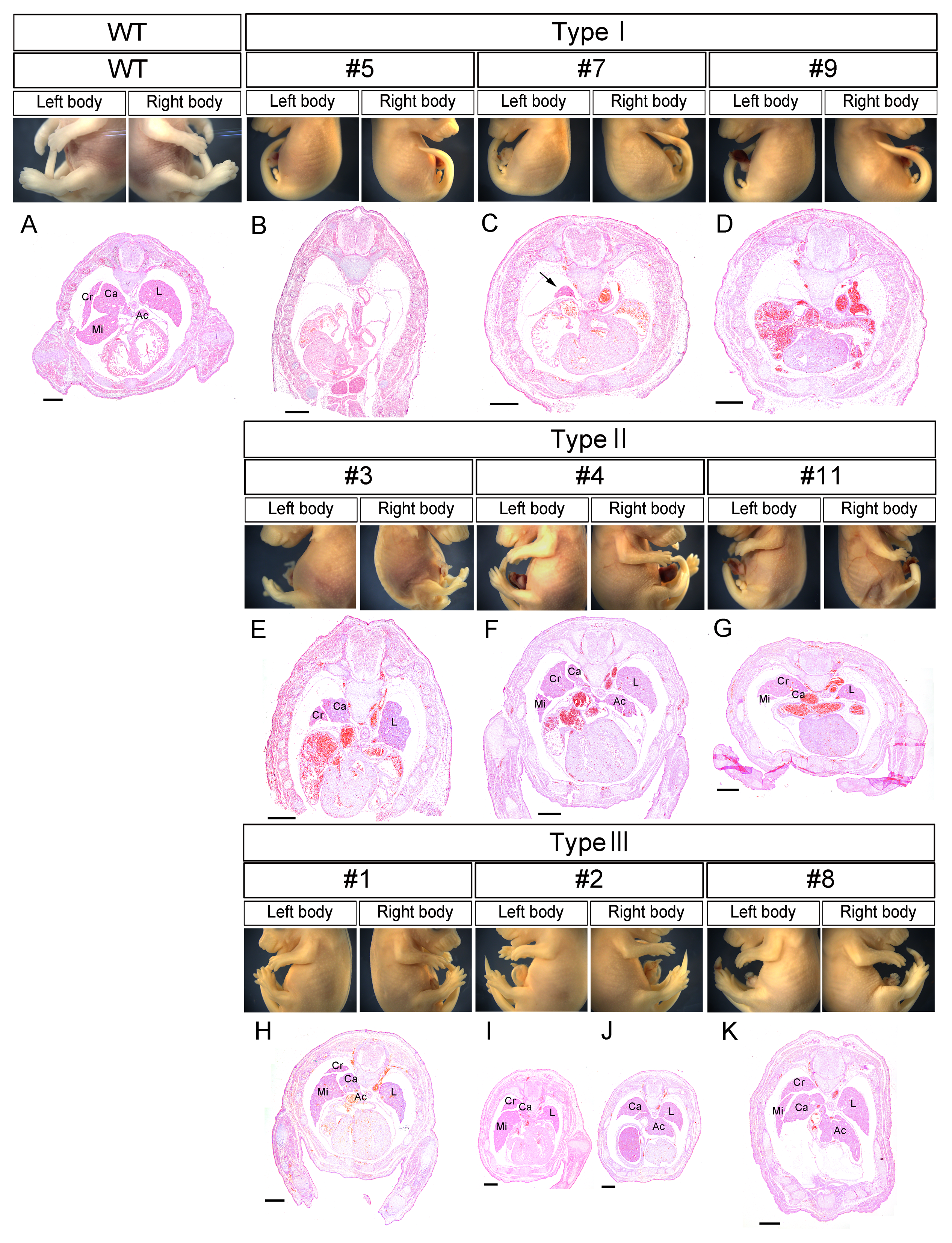

Supplement: S4 Fig — Scale bars: 500 μm. (TIF) [file pone.0240333.s008.tif]

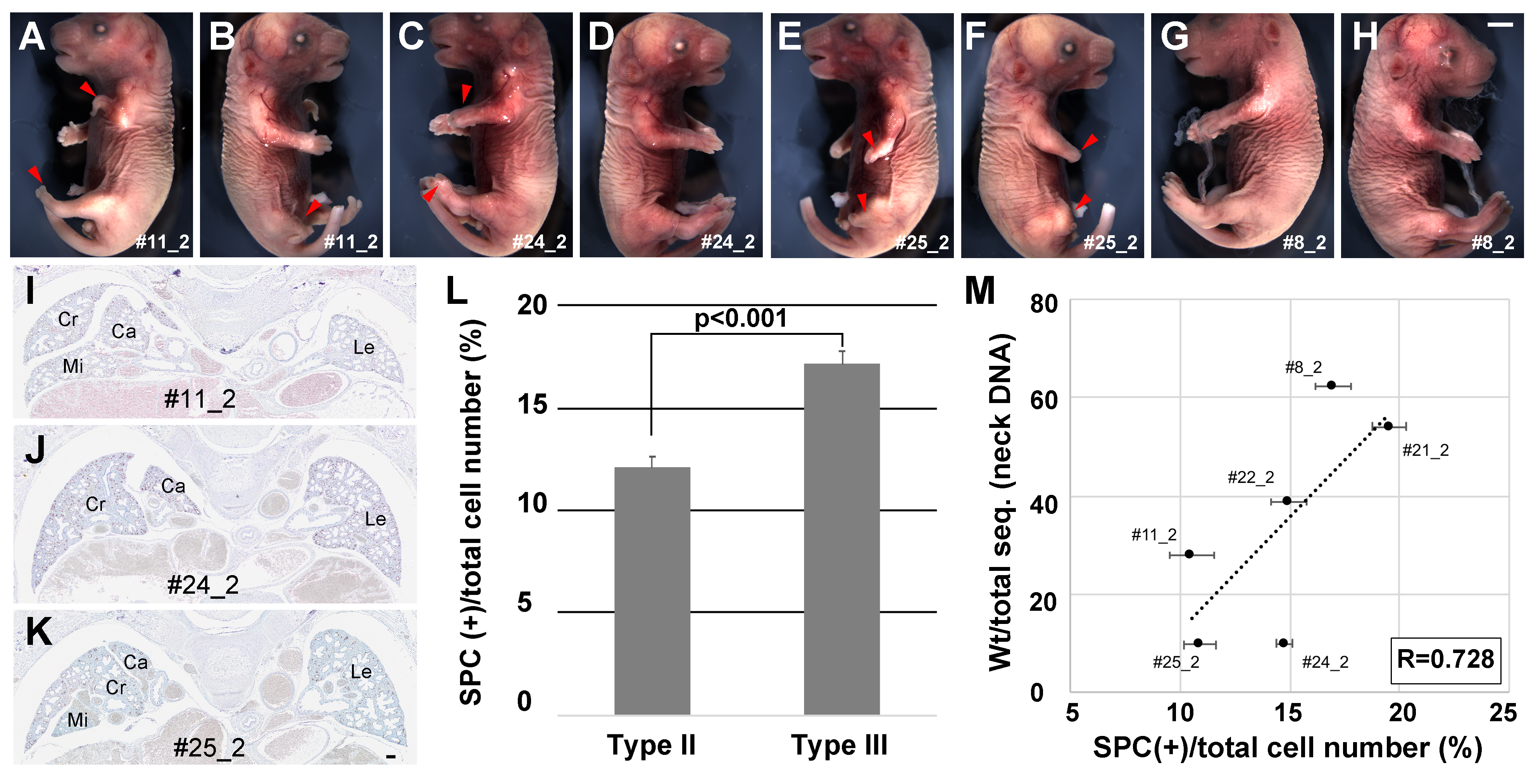

Supplement: S5 Fig — Source data for (L, M) are available in S3 Table. Lateral views of type II (A-F), and type III (G, H) embryos at E18.5. Arrowheads show limb defects. (I-K) In all three type II embryos, the accessory lobe was lost. In embryo #24_2, (J), the middle lobe (Mi) was also undetectable. Data in (L) are presented as means ± SEM. (M) In these embryos, the number of SPC-positive cells was more correlated to the percentage of wild type Fgf10 genotype (correlation coefficient [R] was 0.728 for neck DNA) than that including in-frame mutations with Lys196 and His201 retained (R = 0.334). Scale bar: 2 mm (A-H). (TIF) [file pone.0240333.s009.tif]

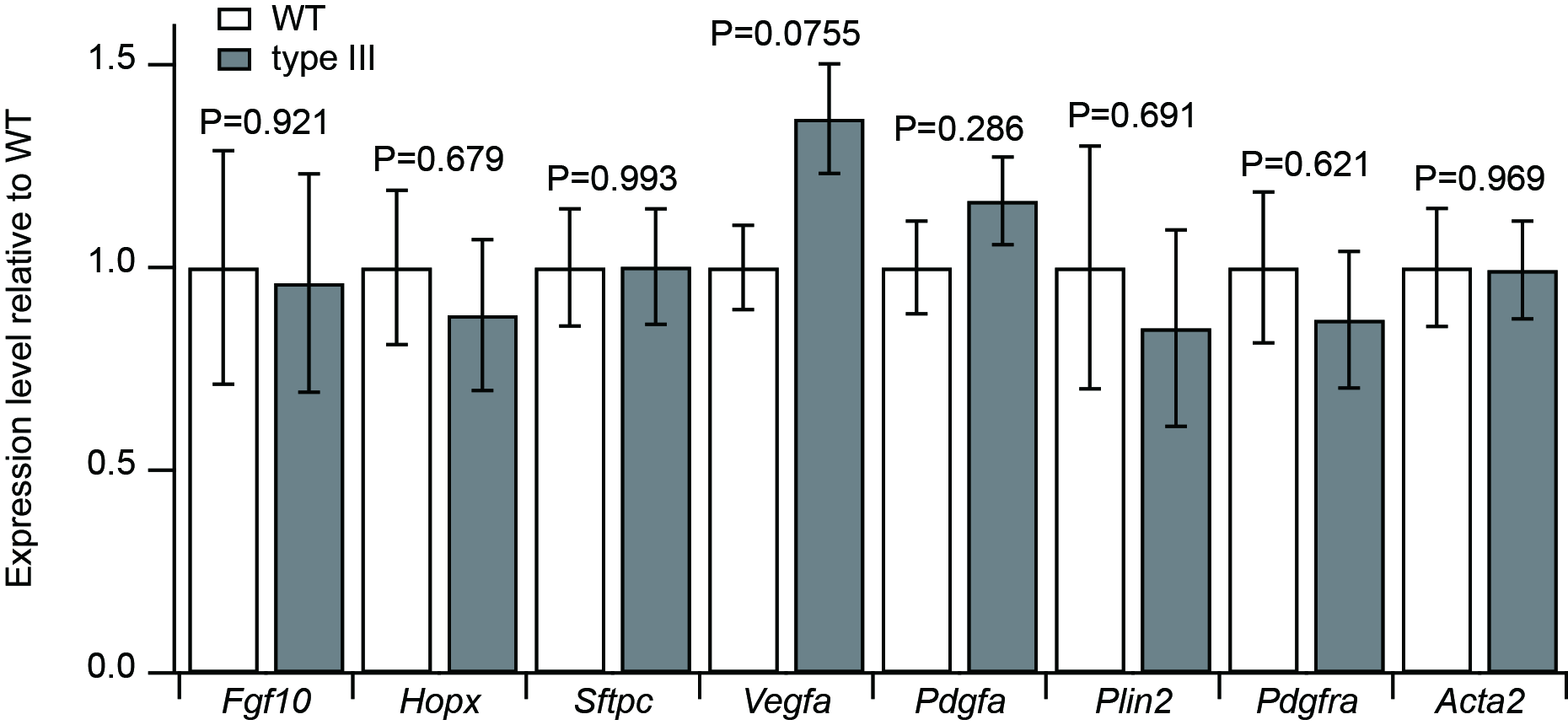

Supplement: S6 Fig — (TIF) [file pone.0240333.s010.tif]

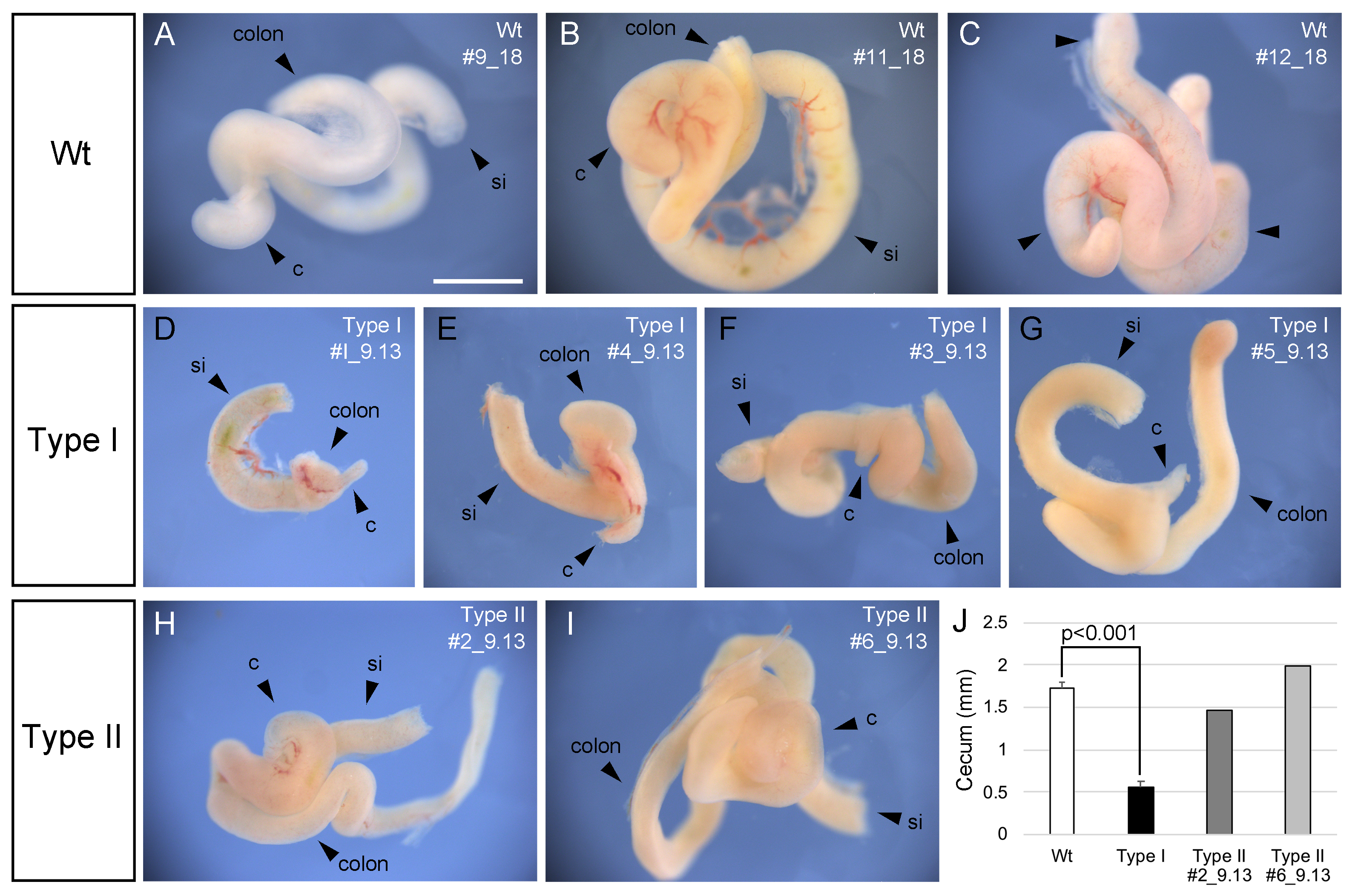

Supplement: S7 Fig — A-C, wild type (Wt) cecum (c), colon, and small intestine (si) are shown from three embryos examined. Ileum and colon were cut at dissection. D-G, type I cecum is reduced compared with the wild type. Whether the cecum epithelium is absent or not cannot be identified from these photos. Type I embryos show an atresia of the colon, but the length varies depending upon the embryos. H-I, type II embryos examined (n = 2) do not exhibit a reduced cecum or an atresia of the colon. The colons presented here were cut as distally as possible. J, the approximate length of the cecum. The length of type I cecum is significantly decreased compared with the wild type. The length of two type II embryos examined is also shown for reference. Scale bar: 1 mm (in A for all to scale). (TIF) [file pone.0240333.s011.tif]
